# Insurance participation, equity pledge and stock price crash risk: Evidence from China

**DOI:** 10.1371/journal.pone.0311219

**Published:** 2024-10-24

**Authors:** Fangjing Hao

**Affiliations:** School of computer science and technology, Shandong Technology and Business University, Yantai, China; The university of Chenab, PAKISTAN

## Abstract

This paper analyzes the causes of equity pledge, external conduction mechanisms, and economic consequences from the perspective of insurance participation by integrating insurance participation, equity pledge, and stock price crash risk into a unified framework. An empirical analysis of sample data from listed companies in Shanghai and Shenzhen between 2007–2021, indicates that equity pledge reduces the risk of collapse as companies hedge the risk induced by the equity pledge. Further research has revealed that insurance participation can mitigate stock price crash risk brought by equity pledge through a regulatory effect, which is more pronounced for private companies and those with a high shareholding ratio, and companies in manufacturing industry. This is because private companies have a higher demand for capital as their property rights are not state-owned, the degree of separation of powers and agency conflicts is greater in companies held by large shareholders, manufacturing companies usually have stable earnings and cash flow performance, and the financial support provided by insurers for equity pledges at risk can effectively reduce the risk of their collapse.

## 1. Introduction

The "Related matters notice of insurance asset management company set up special products" was published on October 25, 2018, allowing insurance asset management companies to set up special products, to maximize the investment advantage of insurance funds over the long term. Additionally, it enables it to support high-quality listed companies and shareholders who possess a competitive technological edge but are temporarily facing liquid difficulties due to equity pledge. Is this policy still a potential minefield, or does it offer a robust guarantee for equity pledge?

The equity pledge has always been a popular approach to financing for shareholders, primarily due to its low financing costs, short time, and high efficiency, however, its risk exposure is exceedingly high. First and foremost, conflicts between managers and shareholders will first force the company to execute equity pledge, based on the principal-agent problem, and as a result of poor corporate governance, a control transfer risk will materialize [1]. Secondly, the "domino effect" may appear, and systematic risk would manifest itself as the share price of the company swings wildly due to the joint pinch of no-change restrictions and the depression linkage effect of the main board market. Consequently, equity pledges are potentially risky for the growth of companies.

However, institutional investors are willing and able to participate actively in corporate governance [2–4]. As institutional investors, insurance companies play a crucial role in corporate governance. Before investing in a listed company, an insurance company will take steps to strengthen its internal governance in order to lessen the likelihood of default on equity pledge. Moreover, its strategic investments in the company provide financial support for the company’s liquidity concerns, which is conducive to a share price increase. However, as institutional investors, insurance companies frequently intervene in the market by manipulating information, and short-term institutional investors focus on short-term profits and reduce the efficiency of their investments [5], thereby burying the bane of the stock price’s decline or even the risk of collapse. In other words, equity pledge is a potential tool for stock price crash risk, but it is uncertain whether insurance participation can effectively mitigate the liquidity risk associated with equity pledge. Accordingly, would the risk of collapse be exacerbated by a lack of solvency, or would there be a shortfall in affordability? This issue merits further investigation.

Based on the preceding analysis, this paper seeks to investigate the primary mechanism of equity pledge from the perspective of insurance participation, conjunction with equity pledge and stock price crash risk; the outcome analysis reveals the following: company that carried out equity pledge experienced low risk of collapse. Further research indicates that insurance participation has a regulatory effect that mitigates the risk of collapse precipitated by equity pledge. Moreover, the effect of lowering crash risk is more pronounced for private companies and those with a high shareholding ratio.

Compared to previous studies, this paper makes the following contributions to research: To start with, despite the fact that there are studies on equity pledge and stock price crash risk, the outcomes are not identical. Consequently, this paper enriches the mechanism of internal conduction between equity pledge and stock price crash risk. Secondly, insurance participation has been the subject of few studies. Seeking to fill the research gap, this paper introduces insurance participation and equity pledge and analyzes their effects on stock price crash risk. This allows for a thorough investigation of equity pledge and stock price in this circumstance and also offers empirical support for the long-term development of the company in the capital market. Third, the participation of insurance funds in stock market investment may exacerbate market volatility, especially large-scale capital inflows into certain stocks or industries may trigger systemic risk, which has a great impact on the risk of collapse, so the research can provide support for the regulatory authorities to formulate regulatory and investment-related policies, in order to safeguard the stability and healthy development of the market.

The remaining sections of this paper are structured as follows: Section 2 examines extensive literature and hypotheses. Section 3 describes the study’s sample and empirical methodology, followed by Section 4, which reports the empirical results. This paper concludes with a discussion of policy implications in section 5.

## 2. Literature review, theoretical analysis and hypotheses development

In the past, studies of the probability of stock price crash risk relied heavily on the principal-agent theory, which posits that managers’ desire for profit is what drives them to conceal negative information about a company’s investment in a project and in turn, causes the stock price to drop [1]. However, it is news, who determine the direction of the market, and the only factor that renders news "good" or "bad" is the way in which it is perceived by the people. In the capital market, good news can generate income for investors, and bad news does the opposite. However, since not every participant in the market is a rational economic person, each investor’s ability to interpret the news varies greatly, so people use the terms bearish and bullish to characterize the news. Generally, bad news refers to the market providing investors with bad earnings headlines, which is a fait accompli. According to the fait accompli of the future extrapolation, analysis, the fact will have a further, more deleterious impact on the future. As a result, investors form more pessimistic expectations, investor confidence in the market decreases, and in order to avoid future negative consequences, they will sell the relevant assets, causing downward share price fluctuations for the company in the market. From this vantage point, it is evident that market does not prefer bad news. However, companies must be able to withstand certain amount of bad news before the share price begins to decline and the risk of collapse increases. When bad news exceeds this threshold, the share price begins to decline, and the risk of collapse increases [6]. Meanwhile disclosure quality problems arising from information asymmetry issues can also cause stock prices to move downward [7], but favorable corporate governance and strict auditing processes can act as a disincentive [8,9]. And an explicit informal hierarchy among directors enhances the coordination of managers’ efforts to conceal bad news, thereby increasing the risk of collapse [10]. And there is a significant negative relationship between government green subsidies and the risk of collapse [11]. Despite the diversity and complexity of the factors influencing the risk of collapse are diverse and complex, equity pledge is also an important factor influencing stock price crash risk.

From the review of equity pledge, previous researchers have focused primarily on the relationship between equity pledge and company value, however, close attention also needs to be given to the analysis of controlling shareholders’ agency conflict-induced encroachment on the interests of minority shareholders [12]. The research of later researchers focuses primarily on analyzing the various aspects of equity pledge and company behavior. Huang and Xue (2016) [13] discovered that a company’s earnings smoothness is greater after equity pledge, and that reduced agency costs increase shareholders’ willingness to pledge equity [14]. And the liquidity in the process affects the risk of collapse [15]. However, the focal point of this paper is on the examination of the relationship between crash risk and equity pledge from the perspective of insurance participation.

As one of the pledge of rights, equity pledge is a leveraged financial transaction in which shareholders mortgage shares of the company. Based on the theory of the financing dilemma, the financing constraints of businesses will lead them to choose the more convenient equity pledge for financing. Enterprises typically improve internal management, anticipate positive market performance, qualify for equity pledge, and use higher leverage, which lowers the cost of financing, to alleviate financial difficulties and prevent financial risks. In addition, since earnings management is an integral part of reflecting information at the corporate level, with corporate performance primarily reflected through earnings management [16], earnings management will influence the attitude of the company’s quality holders toward financing. In other words, the earnings management of listed companies is pivotal. Additionally, the accuracy and transparency of information concerning listed companies can be improved at the level of earnings management, and good resources can be directed toward higher-level earnings companies, which lowers the financing costs of businesses by preventing unnecessary losses and enhances the efficiency of capital market allocation. The allocation of capital market resources is more efficient when more information is available at the company level. Therefore, when companies pledge equity, earnings management will be strengthened, and the company’s overall performance will improve. Good market capitalization management also contributes to the creation of a favorable external financing environment for the company by reducing the "tunnel effect" of controlling shareholders on the interests of the company by controlling shareholders’ squeezing of minority shareholders [12]. Diversity among directors can also reduce the risk of collapse [10], and companies with overconfident CEOs face a higher risk of collapse [17]. In addition, analysts are more optimistic about companies whose controlling shareholders pledge a greater proportion of their shares for bank loans [18]. Moreover, investors in the market will strengthen their recognition of the company, and increase their purchase of its shares, which will go a long way toward boosting the company’s share price yields and reducing its crash risk. Therefore, equity pledge reduces the risk of collapse [19]. Consequently, the H1A hypothesis is proposed.

H1A: Other things being equal, equity pledges and stock price crash risk are negatively correlated.

On the other hand, based on the theory of behavioral finance and the theory of signal transmission, the controlling shareholder may be exposed to the risk of no additional guarantee, and thus the transfer of control rights, which will have a significant impact on the level of corporate governance and the governmental attitude of the controlling shareholders. In order to avoid the risk of close positions and blow-ups, businesses will undoubtedly enhance their earnings management. Furthermore, under the equity pledge, controlling shareholders do not take substantive actions for personal profit. However, this type of corporate management is likely a temporary opportunistic strategy and face-saving process that is neither conducive to the company’s growth nor does it strengthen the internal governance of the company. High-quality earnings management only makes information more opaque, and covers up bad news [20], undermines the debt-binding role that accounting information can play, and asymmetric information reduces investor protection [16]. In addition, Morck et al.(2000) [21] revealed that the content information at the corporate-level is highly limited in emerging capital markets, implying that share prices cannot effectively convey information about earnings management in corporations. Additionally, China is an emerging capital market, which has exacerbated the problem of information asymmetry as a result of the dominance of noise traders. In this instance, the agent conflict within the company’s internal controlling shareholders has intensified. And when the market value of pledged shares falls below the pledgee’s requirements, borrowers must meet margin call requirements, and high-pledge companies are more likely to buy back shares, particularly after a sharp decline in share prices. Otherwise, the pledgee may sell shares to deplete the company’s resources [22]. Likewise, the political dynamics within the organization will lessen the effectiveness of corporate governance [12]. Management’s political expediency stemming from opportunistic speculative motives will only worsen the organization’s operational challenges and lead to stock price fluctuations which are the primary cause of crash risk [23].

Restrictions on equity pledges improve investment efficiency, primarily through the reduction of overinvestment, while equity pledges increase investment risk [24]. This primarily increases the cost of equity capital through information risk and agency conflict channels, and control transfer risk and disclosure costs also increase [25], thereby increasing investor perceptions of risk [26]. Transactions involving the equity pledge can result in investor sentiment bias, which influences investment decisions. Specifically, stock market volatility increases as optimistic and pessimistic investor sentiment toward equity pledge transactions increases [27]. Correspondingly, after shareholders pledge their shares for personal loans, a company’s environmental, social and governance (ESG) performance and the quality of its ESG disclosures significantly decline [28]. Pledging firms also exhibit a significantly higher risk of collapse than non-pledging firms [29]. At the same time, equity pledges can exacerbate the degree of financialization of firms [30], which can also indirectly increase the risk of corporate debt default. Therefore, it is believed that equity pledge will increase the company’s stock price crash risk. Consequently, H1B is proposed:

H1B: When all other conditions are held constant, equity pledge and stock price crash risk are positively correlated.

As important institutional investors, insurance companies are motivated and capable of monitoring the company’s investment and financing practices because they hold equity [31]. Consequently, their desire to prevent management from engaging in opportunistic investment practices and reduce inefficient investments is stronger. In order to help businesses perform better, institutional monitoring restricts managers’ access to the company’s cash flow, which reduces the firm-specific risks that managers must assume [32]. Additionally, due to the different responsibilities that institutional investors engage in as controlling and minority shareholders, they have the ability to supervise the major shareholders and management of businesses using their own financial resources in a manner consistent with the interests of minority shareholders. This contributes to improving corporate governance and enhancement of enterprise value [33]. Institutional investors are, therefore, an effective external governance mechanism, that can effectively mitigate stock price crash risk. The insurance participation of institutional investors can also communicate the internal information of the enterprise to external creditors [34]. Besides, the shareholding percentage of institutional investors also has a significant moderating effect on stock price crash risk [35]. In addition, insurance companies, as stable institutional investors, are more willing to participate in corporate governance, have superior information mining capabilities, and are more concerned with enduring profitability from long-term investment considerations, thereby resolving the investment problem when companies are capital constrained. Therefore, insurance participation can ameliorate the situation of information asymmetry, boost the confidence of financial institutions in the enterprise, and lighten the company’s financing constraints. Their professional and informational advantages prompt the financial market to give heed to their equity participation, which benefits the controlling shareholders by enabling them to carry out equity pledge and draw in more investment. In addition, insurance participation can provide timely financial support to the company to prevent a decline in the stock price in the event of a change in control and an increase in the risk of close position and blow-ups. Moreover, the greater the percentage of insured individuals, the greater the impact can be. This leads to the competing hypothesis:

H2A: Insurance participation serves to diversify the liquidity risk associated with equity pledges, and the greater the participation rate, the less likely the risk of collapse would occur.

Nonetheless, different types of institutional investors have distinct characteristics, with short-term institutional investors focusing on short-term profits, which can decrease the investment efficiency of a company [5]. From a risk-reward perspective, risk-taking increases as earnings expectations rise and decreases as risk expectations fall [36]. The "extraordinary growth" of institutional investors has not made the market more rational, rather, it has increased market risk. Instances in which institutional investors are motivated solely by speculative needs, render them incapable of long-term governance. They typically do not participate in corporate governance, and if the enterprise’s performance is unfavorable, they will choose to "vote with their feet" and leave. Moreover, the greater the institutional shareholding, the greater the degree of information asymmetry manifests, opaque financial reporting also increases the risk of institutional investors’ expectations for future share prices [37], which are more likely to trigger the share price to fall [38]; the "herd effect" will also raise the risk of collapse. When investing based on the interaction between information and psychological behavior, insurance companies and listed companies will conceal bad news when they are communities for their own gain.

The competitive hypothesis is thus presented:

H2B: Insurance participation is not conducive to the liquidity risk of dispersing equity pledge, and the greater the share of equity, the greater the risk of collapse can occur.

Furthermore, companies have different property rights. The Chinese government owns a substantial proportion of listed companies. Chinese state-owned listed companies have excellent access to governmental financial support and commercial contracts, so they have adaptable budget constraints, multiple financing options, and the ability to finance with relative ease, which reduces their reliance on capital market funding [39].

According to Kothari and Wysocki (2009) [40], private companies are less likely than SOEs to have their IPOs approved by the SEC, and are also less likely to raise money through share placements concerning equity financing. When it comes to debt financing, private companies are more costly and have smaller, shorter maturities than SOEs [41]. As a comparatively simple and efficient method of obtaining financing, equity pledges are gaining momentum among private companies. Secondly, from the perspective of institutional arrangement, equity pledges by major shareholders of SOEs are more restrictive and difficult to operate, as they are subject to a number of legal constraints, such as the Company Law and the Guarantee Law, as well as strict internal control and accountability of the company. Therefore, the operational risk of equity pledges by state-owned enterprises is relatively small, and the pressure of margin calls and repayments is relatively small if the share price drops after the equity pledge, there is a synergistic effect of interests between shareholders of state-controlled listed companies and the company, the governance effect is relatively apparent, and the risk of collapse is moderately limited. When the guarantee is insufficient, the stock price is more likely to decline, and private businesses have a difficult time getting financing and managing market risk. Thus, the intervention of insurance participation would provide financial support at a critical time, which facilitates the guarantee increase and investment in stocks, thereby countering the downward trend in share prices and reducing the risk of collapse.

Therefore, the following hypotheses are formulated:

H3: The stock price crash risk of private enterprises is greater than that of state-owned enterprises and the reduction effect of stock price crash risk is more pronounced in the case of insurance participation in private enterprises.

According to the principal-agent theory, major and minor shareholders will decide the company’s development objectives in order to advance their own interests as rational homo economicus. Due to the split between control rights and cash flow rights, dispersed minority shareholders may only hold a fraction of the cash flow rights, and large shareholders of strong shares may also have a leading position by an absolute amount of cash flow rights, allowing them to stifle the voices of small and medium-sized shareholders and force them out of their interests under the trend of concentration of equity. The control of major shareholders remains unchanged, but cash flow is minimal, especially after equity pledge. Greater separation exists between the two powers, and the proxy conflict is more evident. Institutional investors will not compete with major shareholders and will instead collude with them in order to advance their own interests [42]. Thus, large shareholders will gain absolute control of the company and will be profit-driven only to cover their rent-seeking behavior through earnings management information manipulation in order to advance their own interests. Once the bad news is disclosed, the share price will be exposed to downside risk due to the company’s mismanagement of its development risk. Therefore, the major shareholders’ equity pledge under financial constraints increased the degree of separation of two powers and proxy conflict, which grants the listed company a stronger motivation and capacity to empty profits, thereby increasing the risk of collapse. There is also the issue of large shareholders wanting to increase guarantees when share prices fall. If there is not enough money to support the stock price, there will be a greater risk, but insurance participation’s intervention at least provides financial support for the company and shareholders, effectively reducing the stock price crash risk. The following hypothesis is therefore proposed.

H4: Stock price crash risk is greater in companies with large shareholder stakes than in companies with small shareholder stakes, and the reduction effect of stock price crash risk is more prominent in companies with large stakes in major shareholders in the case of insurance participation.

In terms of stock price performance, the performance of companies in different industries varies. Manufacturing companies and technology companies, as different types of industries, also have significant differences in share price performance, and are influenced by the market, industry development and investor preferences. Manufacturing companies typically have stable earnings patterns and cash flows, which allow them to maintain relatively stable stock price performance during fluctuations in the economic cycle. Investors may prefer to buy shares of such companies as they usually provide reliable dividend income and stable long-term growth prospects. Companies are also more likely to be favored by insurers in the case of equity pledges, which can provide timely liquidity to the company in the event of a pledge risk and reduce the risk of its collapse. Technology companies, on the other hand, attract investors mainly due to their innovativeness and possible growth potential, high share price volatility and riskiness, relatively small investor base, high crash risk in the event of increased market uncertainty, coupled with low participation of insurers, the mitigating effect of the company’s crash risk is weaker. The following hypothesis is therefore proposed.

H5: The reduction effect of stock price crash risk is more prominent in companies in manufacturing industry than in technology industry in the case of insurance participation.

## 3. Sample and empirical methodology

### 3.1 Sample

This paper selects as its research object the companies listed on the A-share markets of Shanghai and Shenzhen from 2007 to 2021. The selection of the starting year as 2007 is premised on the policy that new accounting standards have been in effect since 2007. In addition, we exclude: (1) financial listed companies; (2) companies marked ST or *ST that year; (3) some companies with missing data; and (4) a sample of less than 30 annual trading weeks in order to accurately estimate the risk of collapse. To exclude the effect of extreme values, all continuous variables were significant at the 5% level, with a total of 24,413 samples. The involved financial data were obtained from the CSMAR database and the RESSET financial database and were cross-checked.

### 3.2 Empirical models

In this paper, the following models are proposed to test the aforementioned hypotheses: models (1) and (4) test the relationship between equity pledge and stock price crash risk; models (2), (3), (5), and (6) test the impact of insurance participation on equity pledge and stock price crash risk. All six models control annual, industry, and firm variables.


$$NCSKEW_{t+1}=\beta_{0}+\beta_{1}IFPLEDGE_{t}+controls+year+indu+firm+\varepsilon$$ (1)

$$\begin{array}{l} NCSKEW_{t+1}=\beta_{0}+\beta_{1}IFPLEDGE_{t}+\beta_{2}IFPLEDGE_{t}*IP_{t}+\beta_{3}IP_{t}+controls+year+indu\\ +firm+\varepsilon \end{array}$$ (2)

$$\begin{array}{l} NCSKEW_{t+1}=\beta_{0}+\beta_{1}IFPLEDGE_{t}+\beta_{2}IFPLEDGE_{t}*SP_{t}+\beta_{3}SP_{t}+controls+year+indu\\ +firm+\varepsilon \end{array}$$ (3)

$$DUVOL_{t+1}=\beta_{0}+\beta_{1}IFPLEDGE_{t}+controls+year+indu+firm+\varepsilon$$ (4)

$$\begin{array}{l} DUVOL_{t+1}=\beta_{0}+\beta_{1}IFPLEDGEt+\beta_{2}IFPLEDGE_{t}*IP_{t}+\beta_{3}IP_{t}+controls+year+indu\\ +firm+\varepsilon \end{array}$$ (5)

$$\begin{array}{l} DUVOL_{t+1}=\beta_{0}+\beta_{1}IFPLEDGE_{t}+\beta_{2}IFPLEDGE_{t}*SP_{t}+\beta_{3}SP_{t}+controls+year+indu\\ +firm+\varepsilon \end{array}$$ (6)

Among them: NCSKEW_t+1_ and DUVOL_t+1_ represent stock price crash risk; IFPLEDGE_t_ represents whether shareholders carry out equity pledge; The IP_t_ represents whether insurance company shares a stake, SP_t_ represents the stake proportion of insurance company’s participation.

### 3.3 Measuring firm-specific crash risk

According to research by Hutton et al. [37] and Kim et al. [43,44], the residual returns for the extended market model regression are:

$$\gamma_{\mathrm{jt}}=\partial_{j}+\beta_{1j}r_{m(t-2)}+\beta_{2j}r_{m(t-1)}+\beta_{3j}r_{mt}+\beta_{4j}r_{m(t+1)}+\beta_{5j}r_{m(t+2)}+\varepsilon_{jt}$$ (7)

*γ_jt_* is the return of stock j at Week t; *γ_mt_* is the return of market index m at week t, allow asynchronous trading by using leads and lags of market index, weekly return of company j at week t is *W_jt_* = ln(1+*ε_jt_*).

According to a study by Chen et al. [45], the probability of a crash is determined by the bias in negative conditional returns, and NCSKEW is used to represent the value, for company j in year t, its NCSKEW is:

$$NCSKEW_{jt}=-[n{\left(n-1\right)}^{3/2}\sum w_{jt}^{3}]/[(n-1)(n-2){\left(\sum w_{jt}^{2}\right)}^{3/2}]$$ (8)

Where n represents the number of trading weeks for a stock in a year, the larger the value, the higher the crash risk would be for the stock.

DUVOL represents the fluctuation of the stock yield, and the calculation method is as follows:

$$DUVOL_{jt}=\log\{\left[(n_{u}-1)\sum_{Down}w_{jt}^{2}]/[(n_{d}-1)\sum_{Up}w_{jt}^{2}\right]\}$$ (9)

In the Formula (9), n_u_ indicates that the number of weeks in which weekly returns of the stock j Wjt are greater than average annual return Wj, and n_d_ indicates the number of weeks that weekly returns of the stock j Wjt are lesser than average annual return Wj. The numerical size of the variable represents the degree of left deviation of the distribution of stock return; the more left the stock return, the greater the risk of a market crash.

### 3.4 Variables

In this paper, two indicators are used to measure the equity pledge of controlling shareholders. *IFPLEDGE* is a dummy variable, while the equity pledge behavior of controlling shareholders in the statistical year is assigned a value of 1; otherwise, a value of 0 is assigned. *IP* indicates insurance company participation, with a value of 1 if insurance participation is present and 0 otherwise. *SP* indicates the participation rate of insurance companies in shares.

The following control variables have been added based on the research of Jin & Myers [6], Hutton et al.[37], Kim et al.[17,43], Chen et al.[39], Yin and Tian [46], Jebran et al.[47,48]: The shareholding ratio of the largest shareholder (*TOPHLD*_*t*_), the property nature (*SOE*_*t*_), the asset-liability ratio (*DTA*_*t*_), the company’s market to book value ratio (*MTB*_*t*_), return on asset ratio (*ROA*_*t*_), asset size (*SIZE*_*t*_), stock trading turnover(*DTURN*_*t*_), discretionary accruals (*ABACC*_*t*_), past returns(*RET*_*t*_), weekly return volatility(*SIGMA*_*t*_), Whether the chairman and the general manager are the same person(*CP*_*t*_), board independence(*IDR*_*t*_), board size(*SVS*_*t*_), lagged dependent variable of NCSKEW_t+1_(*NCSKEW*_*t*_), lagged dependent variable of DUVOL_t+1_ (*DUVOL*_*t*_), the main variables are interpreted in Table 1.

**Table 1:** Interpretation of main variables.

Variable name	Variable description		
**NCSKEW**_**t+1**_	The negative yield bias coefficient indicates the likelihood that the stock price crash risk occur, and the greater the value, the greater the likelihood that the stock price crash risk would occur		
**DUVOL**_**t+1**_	The fluctuation ratio of annual earnings indicates the likelihood that stock price crash risk occurs, and the greater the value, the greater the likelihood that stock price crash risk would occur		
**IFPLEDGE**_**t**_	Whether the pledge, if there is a pledge of the controlling shareholder within the year, is counted as 1, otherwise 0		
**IP**_**t**_	Whether the insurance company participation, if insurance company participation, is 1, otherwise 0		
**SP**_**t**_	Shareholding ratio of insurance companies		
**TOPHLD**_**t**_	Shareholding ratio of the largest shareholder		
**SOE**_**t**_	The nature of the largest shareholder’s equity properity, if it is the state-owned equity then assignment is 1, otherwise 0		
**DTA**_**t**_	Asset-liability ratio = Total liabilities at end of period/total assets at end of period		
**MTB**_**t**_	Market value of equity divided by book value of equity		
**ROA**_**t**_	Return on assets = EBIT/total assets at the end of the period		
**SIZE**_**t**_	Asset size, natural logarithm of total assets at the end of the period		
**DTURN**_**t**_	It is measured as the difference of mean monthly share turnover of year T and year T-1.		
**ABACC**_**t**_	Absolute value of residuals from the modified Jones model.		
**RET**_**t**_	Average weekly stock returns during year.		
**SIGMA**_**t**_	Standard deviation of weekly stock returns during year		
**CP**_**t**_	If the general manager and the chairman of the board are one person, then 1, otherwise 0		
**IDR**_**t**_	Independent director ratio, which refers to the ratio of independent directors to the size of the board of directors		
**SVS**_**t**_	Board size, mainly referring to the number of board members		
**NCSKEW**_**t**_	lagged dependent variable of NCSKEW_t+1_		
**DUVOL**_**t**_	lagged dependent variable of DUVOL_t+1_		
**YEAR**	Dummy variable of year		
**INDU**	Dummy variable of industry		
**FIRM**	Dummy variable of firm		

## 4. Empirical results

### 4.1 Descriptive statistics

Table 2 is a descriptive statistic of the main variables.

**Table 2:** Descriptive statistics of main variables.

Statistic	Min	Pctl(25)	Median	Pctl(75)	Max	Mean	St. Dev.
**NCSKEW**_**t+1**_	-1.569	-0.683	-0.247	0.142	0.835	-0.285	0.623
**DUVOL**_**t+1**_	-0.995	-0.513	-0.191	0.127	0.607	-0.192	0.439
**IP**_**t**_	0	0	0	1	1	0.257	0.437
**SP**_**t**_	0	0	0	0.0004	0.016	0.002	0.004
**IFPLEDGE**_**t**_	0	0	1	1	1	0.623	0.485
**PRATIO**_**t**_	0	0	0.063	0.182	0.444	0.112	0.134
**TOPHLD**_**t**_	0.135	0.23	0.326	0.45	0.622	0.346	0.141
**SOE**_**t**_	0	0	0	1	1	0.395	0.489
**MTB**_**t**_	0.979	1.243	1.625	2.332	4.64	1.96	0.984
**DTA**_**t**_	0.11	0.271	0.431	0.59	0.784	0.434	0.198
**ROA**_**t**_	-0.054	0.028	0.052	0.083	0.153	0.055	0.049
**SIZE**_**t**_	20.347	21.218	21.972	22.9	24.621	22.13	1.181
**DTURN**_**t**_	-0.02	-0.004	-0.00004	0.004	0.018	-3E-04	0.009
**ABACC**_**t**_	0.001	0.004	0.011	0.029	0.085	0.021	0.024
**RET**_**t**_	-0.012	-0.004	0.002	0.009	0.025	0.004	0.01
**SIGMA**_**t**_	0.033	0.047	0.06	0.078	0.115	0.065	0.022
**CP**_**t**_	0	0	0	1	1	0.265	0.442
**IDR**_**t**_	0.333	0.333	0.333	0.429	0.5	0.373	0.049
**SVS**_**t**_	0	3	3	4	15	3.584	1.117
**NCSKEW**_**t**_	-1.569	-0.682	-0.247	0.143	0.835	-0.284	0.622
**DUVOL**_**t**_	-0.995	-0.512	-0.19	0.126	0.606	-0.192	0.438

As observed in Table 2, the agent variables of stock price crash risk, NCSKEW_t+1_ and DUVOL_t+1_’s standard deviations are 0.623 and 0.439, respectively; the difference is substantial, indicating that the heterogeneity of stock price crash risk on the capital market is very different, and the mean value is more severe by the extremum. The standard deviation of IFPLEDGE_t_ is 0.485, the mean value of PRATIO_t_ of controlling shareholders’ equity pledge ratio is 0.112, and the median value is 0.063, indicating that the heterogeneity of listed companies’ equity pledge behavior and equity pledge ratio varies considerably. The standard deviation of SP_t_ is 0.004, and its maximum and minimum values are 0.016 and 0.000, respectively, suggesting that the heterogeneity of insurance company participation behavior and insurance participation ratios varies considerably.

In addition, descriptive statistics for the majority of the control variables were consistent with prior research. In our sample, approximately 37.3% of board members are independent, and 26.5% of CEOs serve as board chairs, which indicates a better internal and external monitoring mechanism for the development of the company. The average ROA_t_ is 0.055, revealing that most companies have a solid operating performance. The average absolute value of discretionary accruals (ABACCT_t_) is 0.021. In a fiscal year, the average firm-specific weekly stock returns (RET_t_) is 0.004. In a fiscal year, the average standard deviation of firm-specific weekly stock returns (SIGMA_t_) is 0.022, which indicates a better robustness of the company’s growth. Furthermore, our sample companies have an average leverage (DTA_t_) of 0.434, size (SIZE_t_) of 22.13, and market-to-book value ratio (MTB_t_) of 1.96, which shows that the basic situation of the company’s development is normal. The above analysis reveals that the different variables are logically similar to the results of the existing studies.

The data were also analyzed for the correlation of the variables, and Table 3 shows the matrix of correlation coefficients, which shows that the correlation relationships between the variables are all relatively low and there is no problem of multicollinearity.

**Table 3:** Correlation coefficient matrix.

	NCSKEW_t+1_	DUVOL_t+1_	IFPLEDGE_t_	IP_t_	SP_t_	TOPHLD_t_	SOE_t_	DTA_t_	MTB_t_	ROA_t_	SIZE_t_	DTURN_t_	ABACC_t_	RET_t_	SIGMA_t_	CP_t_	IDR_t_	SVS_t_	NCSKEW_t_	DUVOL_t_
NCSKEW_t+1_	1	0.88	0.01	0.03	0.03	-0.01	-0.05	-0.04	0.12	0.08	-0.07	-0.04	-0.04	0.12	0.01	0.03	0.003	-0.02	0.07	0.06
DUVOL_t+1_	0.88	1	0.01	0.03	0.03	-0.02	-0.05	-0.05	0.11	0.07	-0.08	-0.04	-0.06	0.11	0.005	0.03	0.004	-0.03	0.07	0.06
IFPLEDGE_t_	0.02	0.01	1	-0.08	-0.08	-0.2	-0.36	0.01	0.06	-0.06	-0.03	0.03	-0.02	-0.02	0.04	0.12	0.06	-0.16	0.01	0.01
IP_t_	0.03	0.03	-0.08	1	0.98	0.08	0.11	0.04	0.02	0.14	0.23	-0.02	0.13	0.01	-0.06	-0.05	-0.01	0.07	0.03	0.02
SP_t_	0.03	0.03	-0.06	0.73	1	0.06	0.11	0.04	0.02	0.14	0.23	-0.01	0.13	0.003	-0.07	-0.06	-0.01	0.07	0.03	0.02
TOPHLD_t_	-0.02	-0.02	-0.2	0.08	0.01	1	0.2	0.03	-0.12	0.13	0.12	-0.02	0.07	-0.001	-0.05	-0.04	0.01	0.07	-0.01	-0.02
SOE_t_	-0.05	-0.05	-0.36	0.11	0.09	0.21	1	0.26	-0.16	-0.09	0.24	-0.02	0.17	0.01	-0.04	-0.29	-0.1	0.4	-0.05	-0.05
DTA_t_	-0.04	-0.05	0.01	0.03	0.03	0.03	0.26	1	-0.31	-0.25	0.4	-0.002	0.36	0.02	0.03	-0.14	-0.04	0.19	-0.04	-0.04
MTB_t_	0.1	0.1	0.05	0.01	0.004	-0.11	-0.13	-0.28	1	0.19	-0.47	-0.05	-0.29	0.4	0.3	0.1	0.04	-0.12	0.01	-0.005
ROA_t_	0.08	0.07	-0.07	0.14	0.1	0.13	-0.08	-0.26	0.17	1	0.06	0.01	-0.001	0.11	-0.09	0.02	-0.04	-0.02	0.05	0.03
SIZE_t_	-0.06	-0.08	-0.04	0.23	0.18	0.14	0.25	0.4	-0.4	0.06	1	0.03	0.62	-0.04	-0.24	-0.15	-0.03	0.2	-0.08	-0.1
DTURN_t_	-0.04	-0.04	0.03	-0.01	0.01	-0.01	-0.02	0.001	-0.04	-0.001	0.03	1	0.02	0.1	-0.24	0.01	-0.004	-0.01	0.04	0.04
ABACC_t_	-0.04	-0.06	-0.03	0.12	0.09	0.09	0.17	0.34	-0.24	-0.03	0.61	0.02	1	-0.04	-0.11	-0.12	-0.01	0.14	-0.06	-0.07
RET_t_	0.12	0.11	-0.02	0.004	-0.02	-0.002	0.02	0.03	0.39	0.11	-0.06	0.07	-0.04	1	0.35	-0.01	-0.004	0.01	-0.15	-0.17
SIGMA_t_	-0.002	-0.01	0.04	-0.06	-0.08	-0.05	-0.03	0.04	0.28	-0.1	-0.23	-0.25	-0.1	0.43	1	0.03	0.02	-0.04	-0.09	-0.09
CP_t_	0.03	0.03	0.12	-0.05	-0.05	-0.04	-0.29	-0.14	0.08	0.02	-0.15	0.005	-0.11	-0.01	0.03	1	0.12	-0.17	0.02	0.03
IDR_t_	0.002	0.004	0.07	-0.01	-0.01	0.01	-0.12	-0.04	0.05	-0.03	-0.04	-0.01	-0.01	-0.01	0.02	0.13	1	-0.11	-0.003	0.002
SVS_t_	-0.02	-0.02	-0.16	0.07	0.05	0.07	0.39	0.19	-0.1	-0.01	0.21	-0.004	0.14	0.02	-0.03	-0.17	-0.12	1	-0.02	-0.02
NCSKEW_t_	0.07	0.06	0.02	0.03	0.04	-0.02	-0.05	-0.04	0.01	0.05	-0.08	0.04	-0.06	-0.13	-0.09	0.02	-0.003	-0.01	1	0.89
DUVOL_t_	0.06	0.06	0.01	0.02	0.04	-0.02	-0.05	-0.05	-0.01	0.03	-0.1	0.04	-0.07	-0.15	-0.08	0.03	0.001	-0.02	0.88	1

Notes: Pearson correlation coefficients are shown in the lower left and Spearman correlation coefficients in the upper right.

### 4.2 Benchmark analysis

Table 4 displays the test results for Eqs (1) through (6). Firstly, the relationship between equity pledges and the stock price crash risk is investigated, and columns (1) through (3) examine the relationship between equity pledges and the risk of collapse. It was determined that the coefficient of IFPLEDGE_t_ in columns (1) and (4) is -0.0178 and -0.0233, respectively, which are all negative and significant at the 10% or 5% level, representing a high negative linear correlation between crash risk and equity pledge and validating hypothesis H1A. This is attributable to the fact that the company will not only improve internal management and performance, but it will also reduce the market circulation of equity. In the case of equity pledge, the market capital supply will exceed the supply of stocks, resulting in fewer tradable shares. As a result, stock prices are resistant to decline, market investors’ trading sentiment is relatively stable and less prone to swing, and stock price crash risk is low in the case of equity pledge, validating hypothesis H1A.

**Table 4:** Test results of the relationship between equity pledge and stock price crash risk.

	NCSKEW_t+1_			DUVOL_t+1_		
	(1)	(2)	(3)	(4)	(5)	(6)
**IFPLEDGE**_**t**_	-0.0178*	-0.0247*	-0.0260*	-0.0233**	-0.0297***	-0.0288***
	(0.0101)	(0.0139)	(0.0147)	(0.0095)	(0.0105)	(0.0101)
**IFPLEDGE**_**t**_ **_IP**_**t**_		-0.0248*			-0.0228*	
		(0.0140)			(0.0129)	
**IP**_**t**_		-0.0040*			-0.0015	
		(0.0023)			(0.0118)	
**IFPLEDGE**_**t**_ **_SP**_**t**_			-3.9506*			-2.6947*
			(2.0897)			(1.4209)
**SP**_**t**_			-0.8656*			-0.0038*
			(0.4894)			(0.0021)
**TOPHLD**_**t**_	-0.0456	-0.0484	-0.0436	-0.0431	-0.0456	-0.0411
	(0.0836)	(0.0837)	(0.0837)	(0.0576)	(0.0577)	(0.0577)
**DTA**_**t**_	-0.1651***	-0.1584***	-0.1588***	-0.0817**	-0.0760**	-0.0760**
	(0.0499)	(0.0500)	(0.0499)	(0.0341)	(0.0341)	(0.0341)
**SOE**_**t**_	-0.0245	-0.0242	-0.0242	-0.0188	-0.0186	-0.0183
	(0.0358)	(0.0358)	(0.0357)	(0.0234)	(0.0235)	(0.0234)
**MTB**_**t**_	0.0776***	0.0764***	0.0769***	0.0528***	0.0519***	0.0520***
	(0.0076)	(0.0076)	(0.0076)	(0.0052)	(0.0052)	(0.0052)
**ROA**_**t**_	0.3453***	0.3454***	0.3472***	0.2080**	0.2082**	0.2096**
	(0.1324)	(0.1324)	(0.1325)	(0.0915)	(0.0915)	(0.0915)
**SIZE**_**t**_	0.0418***	0.0378***	0.0392***	0.0127	0.0094	0.0101
	(0.0132)	(0.0133)	(0.0133)	(0.0091)	(0.0092)	(0.0092)
**DTURN**_**t**_	-5.8103***	-5.7886***	-5.8137***	-4.3044***	-4.2867***	-4.3089***
	(0.5047)	(0.5046)	(0.5046)	(0.3486)	(0.3485)	(0.3486)
**ABACC**_**t**_	0.4113	0.4215*	0.4202*	0.3132*	0.3218*	0.3210*
	(0.2520)	(0.2519)	(0.2520)	(0.1730)	(0.1729)	(0.1729)
**RET**_**t**_	5.0768***	5.1022***	5.1042***	3.3843***	3.4039***	3.4186***
	(0.5320)	(0.5315)	(0.5322)	(0.3723)	(0.3720)	(0.3725)
**SIGMA**_**t**_	-3.4483***	-3.4430***	-3.4461***	-2.7528***	-2.7489***	-2.7486***
	(0.2371)	(0.2372)	(0.2371)	(0.1661)	(0.1662)	(0.1661)
**CP**_**t**_	0.0231	0.0231	0.0230	0.0058	0.0058	0.0057
	(0.0160)	(0.0159)	(0.0159)	(0.0108)	(0.0108)	(0.0108)
**IDR**_**t**_	-0.0400	-0.0441	-0.0425	-0.0173	-0.0206	-0.0204
	(0.1466)	(0.1465)	(0.1466)	(0.1014)	(0.1014)	(0.1014)
**SVS**_**t**_	-0.0201*	-0.0199*	-0.0199*	-0.0141*	-0.0139*	-0.0138*
	(0.0104)	(0.0103)	(0.0104)	(0.0076)	(0.0075)	(0.0075)
**NCSKEW**_**t**_	-0.0967***	-0.0968***	-0.0970***			
	(0.0071)	(0.0070)	(0.0071)			
**DUVOL**_**t**_				-0.0602***	-0.0603***	-0.0604***
				(0.0049)	(0.0049)	(0.0049)
**_cons**	41.5072***	40.3165***	40.7705***	22.7722***	21.8056***	22.0307***
	(4.2644)	(4.3057)	(4.2787)	(2.9027)	(2.9342)	(2.9159)
**Industry**	YES	YES	YES	YES	YES	YES
**Year**	YES	YES	YES	YES	YES	YES
**Firm**	YES	YES	YES	YES	YES	YES
**R** ^**2**^	0.0402	0.0404	0.0405	0.0370	0.0374	0.0374
***N***	24413	24413	24413	24413	24413	24413

Notes: For each variable, we report the regression coefficient and t-statistic (in parentheses) calculated

***,**, and * represent 1%, 5%, and 10% significant levels.

The regression results of Eqs (2), (3), (5) and (6), show that the coefficients of IFPLEDGE_t_ are negative and significant at 10% or 1% level, and the interaction of IFPLEDGE_t_ and SP_t_ (IFPLEDGE_t__SP_t_) and the interaction of IFPLEDGE_t_ and IP_t_ (IFPLEDGE_t__IP_t_) are negative and significant at 10% level, which validates the hypothesis H2A. This indicates that insurance participation further reduces the stock price crash risk through equity pledge. The reason for this is that when a company carries out equity pledge, having an insurance policy in place not only helps to reduce the risk of default, but also improves the company’s internal management and oversight. In addition, the company can use the equity pledge to provide financial support in the event of a liquidity shortage risk and prevent share prices from experiencing a downward trend. Investors’ stable sentiment also reduces the "herd effect", thereby reducing the risk of collapse, thus validating the H2A hypothesis.

### 4.3 Further testing

Table 5 reports the regression results of Eqs (1)–(6) for private enterprises and state-owned enterprises. The coefficients of IFPLEDGE_t_ in private enterprise samples are all negative and statistically significant at the 1%, 5%, and 10% levels, respectively, according to the regression results of columns (1) through (12). And the coefficients of the interaction of IFPLEDGE_t_ and SP_t_ (IFPLEDGE_t__SP_t_) and the interaction of IFPLEDGE_t_ and IP_t_ (IFPLEDGE_t__IP_t_) are also negative and significant at the 10% level, whereas the coefficients in the samples of state-owned enterprises are not significant, reflecting that the effect of reducing stock price crash risk through equity pledge is more prominent in private enterprise samples in the case of insurance participation. State-owned companies are highly protected by the government due to its property rights, and share prices fluctuate more smoothly as a result. And private enterprises initially face greater financing constraints. As a result, a large amount of equity will be pledged, and the risk threshold will also rise. Once the risk of closing a position or blow-ups is encountered, stock price crash risk will rapidly increase. However, insurance participation can strengthen the oversight of private companies, improve the quality of their governance, and bolster market confidence, allowing investors to be optimistic about the company’s growth prospects, thereby increasing stock purchases. Thus, stock price crash risk can be effectively mitigated. The H3 hypothesis is thereby validated.

**Table 5:** Test results of the sample of private enterprises and state-owned enterprises.

	State-owned enterprises						Private Companies					
	NCSKEW _t+1_			DUVOL _t+1_			NCSKEW _t+1_			DUVOL _t+1_		
	(1)	(2)	(3)	(4)	(5)	(6)	(7)	(8)	(9)	(10)	(11)	(12)
**IFPLEDGE**_**t**_	0.0036	-0.0143	-0.0077	0.0022	-0.0041	-0.0066	-0.0031*	-0.0499**	-0.0473**	-0.0431*	-0.0428***	-0.0394***
	(0.1132)	(0.0224)	(0.0211)	(0.0015)	(0.0156)	(0.0146)	(0.0017)	(0.0218)	(0.0208)	(0.0230)	(0.0149)	(0.0144)
**IFPLEDGE**_**t**_ **_IP**_**t**_		-0.0053			0.0013			-0.0420*			-0.0326*	
		(0.0318)			(0.0220)			(0.0218)			(0.0151)	
**IFPLEDGE**_**t**_ **_SP**_**t**_			1.8211			1.0899			-4.5169*			-2.6718*
			(2.9674)			(2.0337)			(2.1904)			(1.3969)
**IP**_**t**_		-0.0258			-0.0143			-0.0135*			-0.0081	
		(0.0212)			(0.0145)			(0.0058)			(0.0206)	
**SP**_**t**_			-0.4539			-0.5843			-1.5860			-0.0803*
			(2.0578)			(1.3425)			(3.2542)			(0.0493)
**TOPHLD**_**t**_	-0.1117	-0.1037	-0.1001	-0.0468	-0.0483	-0.0458	-0.1199	-0.1286	-0.1229	-0.1379*	-0.1455*	-0.1402*
	(0.1330)	(0.1329)	(0.1330)	(0.0917)	(0.0923)	(0.0924)	(0.1155)	(0.1158)	(0.1157)	(0.0794)	(0.0797)	(0.0797)
**DTA**_**t**_	-0.0914	-0.0834	-0.0856	-0.0135	-0.0100	-0.0113	-0.1507**	-0.1430**	-0.1431**	-0.0871*	-0.0804*	-0.0799*
	(0.0793)	(0.0793)	(0.0792)	(0.0548)	(0.0548)	(0.0547)	(0.0659)	(0.0660)	(0.0658)	(0.0450)	(0.0450)	(0.0449)
**MTB**_**t**_	0.0607***	0.0581***	0.0598***	0.0454***	0.0439***	0.0447***	0.0885***	0.0889***	0.0891***	0.0578***	0.0580***	0.0580***
	(0.0133)	(0.0133)	(0.0133)	(0.0092)	(0.0092)	(0.0092)	(0.0093)	(0.0093)	(0.0093)	(0.0064)	(0.0064)	(0.0064)
**ROA**_**t**_	0.3498	0.3414	0.3476	0.2152	0.2125	0.2156	0.3130*	0.3069*	0.3078*	0.1901	0.1846	0.1861
	(0.2214)	(0.2216)	(0.2216)	(0.1507)	(0.1508)	(0.1508)	(0.1672)	(0.1673)	(0.1673)	(0.1167)	(0.1167)	(0.1166)
**SIZE**_**t**_	-0.0041	-0.0104	-0.0072	-0.0128	-0.0159	-0.0143	0.0776***	0.0773***	0.0783***	0.0298**	0.0295**	0.0299**
	(0.0213)	(0.0215)	(0.0215)	(0.0149)	(0.0150)	(0.0150)	(0.0175)	(0.0179)	(0.0177)	(0.0120)	(0.0122)	(0.0122)
**DTURN**_**t**_	-5.5525***	-5.5337***	-5.5677***	-3.6807***	-3.6514***	-3.6738***	-5.9976***	-5.9463***	-5.9749***	-4.6394***	-4.5944***	-4.6225***
	(0.8470)	(0.8469)	(0.8470)	(0.5953)	(0.5957)	(0.5965)	(0.6313)	(0.6312)	(0.6315)	(0.4329)	(0.4331)	(0.4332)
**ABACC**_**t**_	-0.2773	-0.2672	-0.2707	-0.1420	-0.1326	-0.1360	0.9009***	0.8940***	0.8947***	0.6704***	0.6645***	0.6672***
	(0.3749)	(0.3749)	(0.3749)	(0.2528)	(0.2529)	(0.2530)	(0.3416)	(0.3414)	(0.3413)	(0.2376)	(0.2377)	(0.2375)
**RET**_**t**_	7.3062***	7.3719***	7.3469***	4.5588***	4.5855***	4.5809***	3.8896***	3.8516***	3.8660***	2.7135***	2.6821***	2.7084***
	(0.8702)	(0.8690)	(0.8700)	(0.6039)	(0.6037)	(0.6050)	(0.6873)	(0.6860)	(0.6872)	(0.4901)	(0.4887)	(0.4893)
**SIGMA**_**t**_	-2.7196***	-2.6995***	-2.7149***	-2.1434***	-2.1291***	-2.1359***	-3.9532***	-3.9282***	-3.9244***	-3.1200***	-3.0976***	-3.0933***
	(0.3572)	(0.3579)	(0.3577)	(0.2548)	(0.2550)	(0.2551)	(0.3169)	(0.3177)	(0.3177)	(0.2211)	(0.2215)	(0.2214)
**CP**_**t**_	0.0630**	0.0629**	0.0627**	0.0315	0.0313	0.0312	0.0119	0.0118	0.0117	-0.0025	-0.0026	-0.0026
	(0.0274)	(0.0274)	(0.0274)	(0.0194)	(0.0194)	(0.0194)	(0.0194)	(0.0194)	(0.0194)	(0.0132)	(0.0132)	(0.0132)
**IDR**_**t**_	0.0076	0.0075	0.0108	-0.0410	-0.0439	-0.0427	-0.0858	-0.0898	-0.0912	-0.0027	-0.0065	-0.0086
	(0.2259)	(0.2256)	(0.2258)	(0.1598)	(0.1597)	(0.1597)	(0.2011)	(0.2011)	(0.2012)	(0.1375)	(0.1375)	(0.1376)
**SVS**_**t**_	-0.0110	-0.0110	-0.0109	-0.0082	-0.0083	-0.0082	-0.0273	-0.0274	-0.0272	-0.0168	-0.0169	-0.0168
	(0.0126)	(0.0125)	(0.0126)	(0.0093)	(0.0092)	(0.0092)	(0.0194)	(0.0194)	(0.0194)	(0.0142)	(0.0143)	(0.0143)
**NCSKEW**_**t**_	-0.0661***	-0.0662***	-0.0665***				-0.1271***	-0.1271***	-0.1272***			
	(0.0117)	(0.0117)	(0.0117)				(0.0088)	(0.0088)	(0.0088)			
**DUVOL**_**t**_				-0.0439***	-0.0439***	-0.0441***				-0.0766***	-0.0767***	-0.0768***
				(0.0079)	(0.0079)	(0.0079)				(0.0062)	(0.0062)	(0.0062)
**_cons**	17.9452***	16.3982***	17.2918***	6.2052	5.3262	5.7705	67.0591***	64.4084***	64.7941***	40.5784***	38.2293***	38.4319***
	(6.0118)	(6.0657)	(6.0387)	(4.0050)	(4.0618)	(4.0310)	(6.4923)	(6.6009)	(6.5416)	(4.4742)	(4.5450)	(4.5131)
**Industry**	YES	YES	YES	YES	YES	YES	YES	YES	YES	YES	YES	YES
**Year**	YES	YES	YES	YES	YES	YES	YES	YES	YES	YES	YES	YES
**Firm**	YES	YES	YES	YES	YES	YES	YES	YES	YES	YES	YES	YES
***R*** ^**2**^	0.0354	0.0357	0.0355	0.0319	0.0321	0.0320	0.0506	0.0512	0.0512	0.0457	0.0466	0.0467
***N***	9299	9299	9299	9299	9299	9299	15114	15114	15114	15114	15114	15114

Notes: For each variable, we report the regression coefficient and t-statistic (in parentheses) calculated

***, **, and * represent 1%, 5%, and 10% significant levels.

Table 6 presents the regression results of a split sample of shareholders of various sizes based on Eqs (1)–(6). The regression results of columns (1) through (12) indicate that the coefficients of IFPLEDGE_t_ in samples of major shareholders are negative and statistically significant at the 5% and 10% levels, respectively. And the interaction coefficients of IFPLEDGE_t_ and SP_t_ (IFPLEDGE_t__SP_t_) and the interaction coefficients of IFPLEDGE_t_ and IP_t_ (IFPLEDGE_t__IP_t_) are negative and significant at the 5% level, whereas the coefficients in the sample of minority shareholders are less significant, indicating that the effect of reducing stock price crash risk is more pronounced in the sample of major shareholders in the case of equity pledge through insurance participation. This is because it is easier for major shareholders to make hollow promises to the company as a result of opportunistic speculation, and insurance participation can effectively monitor and prevent speculation from occurring. In addition, equity pledges by major shareholders render it easier to add leverage to financing, which increases the transfer risk of corporate control. And insurance participation helps to provide companies with support when they face security shortfalls in guarantee in the event that they hit the warning line of close position and blow up, prevent share prices from falling, and reduce the risk of collapse, validating the H4 hypothesis.

**Table 6:** Test results of split sample of different shareholder size.

	Sample of Major shareholders						Sample of Small shareholders					
	NCSKEW _t+1_			DUVOL _t+1_			NCSKEW _t+1_			DUVOL _t+1_		
	(1)	(2)	(3)	(4)	(5)	(6)	(7)	(8)	(9)	(10)	(11)	(12)
**IFPLEDGE**_**t**_	-0.0268	-0.0416*	-0.0338*	-0.1532*	-0.0338**	-0.0265*	-0.0451	-0.0198	-0.0256	-0.0254	-0.0327**	-0.0348**
	(0.0203)	(0.0240)	(0.0200)	(0.0907)	(0.0165)	(0.0157)	(0.0348)	(0.0214)	(0.0205)	(0.0283)	(0.0147)	(0.0142)
**IFPLEDGE**_**t**_ **_IP**_**t**_		-0.0711**			-0.0517**			-0.0076			0.0052	
		(0.0308)			(0.0209)			(0.0325)			(0.0227)	
**IFPLEDGE**_**t**_ **_SP**_**t**_			-7.0126**			-4.3661**			1.7533			1.6477
			(3.1145)			(2.1018)			(3.0792)			(2.1379)
**IP**_**t**_		-0.0059			-0.0072			0.0126			0.0070	
		(0.0225)			(0.0152)			(0.0285)			(0.0197)	
**SP**_**t**_			-1.9145			-0.5896			0.0674			0.4382
			(2.3145)			(1.4903)			(2.7369)			(1.8389)
**TOPHLD**_**t**_	-0.1459	-0.1548	-0.1443	-0.0365	-0.0451	-0.0371	0.2699	0.2573	0.2555	0.1426	0.1229	0.1231
	(0.1879)	(0.1889)	(0.1887)	(0.1257)	(0.1263)	(0.1263)	(0.2164)	(0.2169)	(0.2171)	(0.1536)	(0.1540)	(0.1540)
**DTA**_**t**_	-0.2640***	-0.2479***	-0.2529***	-0.1264**	-0.1155**	-0.1183**	-0.0931	-0.0925	-0.0895	-0.0364	-0.0345	-0.0324
	(0.0841)	(0.0842)	(0.0842)	(0.0566)	(0.0565)	(0.0566)	(0.0698)	(0.0699)	(0.0697)	(0.0483)	(0.0482)	(0.0481)
**MTB**_**t**_	0.0696***	0.0687***	0.0693***	0.0452***	0.0448***	0.0448***	0.0876***	0.0878***	0.0877***	0.0616***	0.0620***	0.0620***
	(0.0117)	(0.0117)	(0.0117)	(0.0081)	(0.0080)	(0.0080)	(0.0109)	(0.0110)	(0.0109)	(0.0075)	(0.0075)	(0.0075)
**ROA**_**t**_	0.2671	0.2570	0.2657	0.1812	0.1747	0.1801	0.3061*	0.3042*	0.3070*	0.2211*	0.2195*	0.2216*
	(0.2179)	(0.2176)	(0.2177)	(0.1460)	(0.1458)	(0.1457)	(0.1798)	(0.1799)	(0.1800)	(0.1268)	(0.1269)	(0.1269)
**SIZE**_**t**_	0.0456*	0.0395*	0.0431*	0.0089	0.0054	0.0069	0.0468**	0.0479**	0.0475**	0.0203	0.0218	0.0216
	(0.0240)	(0.0239)	(0.0240)	(0.0159)	(0.0158)	(0.0159)	(0.0194)	(0.0199)	(0.0197)	(0.0136)	(0.0139)	(0.0139)
**DTURN**_**t**_	-6.6619***	-6.6185***	-6.6622***	-4.9341***	-4.9022***	-4.9350***	-5.5084***	-5.4730***	-5.4805***	-4.0588***	-4.0067***	-4.0193***
	(0.8794)	(0.8794)	(0.8789)	(0.6231)	(0.6236)	(0.6233)	(0.6349)	(0.6346)	(0.6349)	(0.4369)	(0.4370)	(0.4374)
**ABACC**_**t**_	0.0698	0.0914	0.0826	0.1445	0.1595	0.1534	0.4757	0.4762	0.4798	0.3798	0.3813	0.3838
	(0.3781)	(0.3773)	(0.3780)	(0.2607)	(0.2606)	(0.2608)	(0.3534)	(0.3538)	(0.3536)	(0.2456)	(0.2458)	(0.2457)
**RET**_**t**_	5.5923***	5.5611***	5.5899***	3.6309***	3.6004***	3.6389***	4.4927***	4.4656***	4.4765***	3.0757***	3.0353***	3.0489***
	(0.8282)	(0.8274)	(0.8275)	(0.5704)	(0.5699)	(0.5704)	(0.7376)	(0.7388)	(0.7395)	(0.5230)	(0.5234)	(0.5237)
**SIGMA**_**t**_	-3.5750***	-3.5748***	-3.5784***	-2.8069***	-2.8063***	-2.8033***	-3.6149***	-3.5961***	-3.5959***	-2.8126***	-2.7871***	-2.7860***
	(0.3636)	(0.3642)	(0.3640)	(0.2551)	(0.2552)	(0.2551)	(0.3277)	(0.3280)	(0.3278)	(0.2297)	(0.2299)	(0.2298)
**CP**_**t**_	0.0055	0.0043	0.0041	-0.0207	-0.0217	-0.0219	0.0346*	0.0348*	0.0347*	0.0204	0.0207	0.0207
	(0.0286)	(0.0285)	(0.0286)	(0.0191)	(0.0191)	(0.0191)	(0.0207)	(0.0207)	(0.0207)	(0.0143)	(0.0143)	(0.0143)
**IDR**_**t**_	-0.0300	-0.0405	-0.0339	0.0495	0.0426	0.0455	-0.0423	-0.0473	-0.0482	-0.0768	-0.0829	-0.0848
	(0.2325)	(0.2320)	(0.2323)	(0.1595)	(0.1594)	(0.1595)	(0.2031)	(0.2031)	(0.2031)	(0.1417)	(0.1416)	(0.1415)
**SVS**_**t**_	-0.0049	-0.0052	-0.0051	-0.0060	-0.0062	-0.0061	-0.0376**	-0.0379**	-0.0378**	-0.0203*	-0.0207*	-0.0207*
	(0.0157)	(0.0156)	(0.0158)	(0.0111)	(0.0110)	(0.0111)	(0.0155)	(0.0155)	(0.0155)	(0.0116)	(0.0116)	(0.0116)
**NCSKEW**_**t**_	-0.1274***	-0.1272***	-0.1274***				-0.1141***	-0.1141***	-0.1142***			
	(0.0112)	(0.0112)	(0.0112)				(0.0094)	(0.0094)	(0.0094)			
**DUVOL**_**t**_				-0.0798***	-0.0796***	-0.0797***				-0.0697***	-0.0697***	-0.0698***
				(0.0076)	(0.0076)	(0.0076)				(0.0066)	(0.0066)	(0.0066)
**_cons**	42.7544***	40.2330***	41.4536***	22.8615***	21.2210***	21.7332***	44.6658***	43.8827***	43.7925***	25.6465***	24.5098***	24.4962***
	(7.0256)	(7.0341)	(7.0375)	(4.6520)	(4.6758)	(4.6722)	(5.9655)	(6.0282)	(5.9909)	(4.1009)	(4.1269)	(4.1031)
**Industry**	YES	YES	YES	YES	YES	YES	YES	YES	YES	YES	YES	YES
**Year**	YES	YES	YES	YES	YES	YES	YES	YES	YES	YES	YES	YES
**Firm**	YES	YES	YES	YES	YES	YES	YES	YES	YES	YES	YES	YES
***R*** ^**2**^	0.0452	0.0463	0.0459	0.0404	0.0416	0.0412	0.0450	0.0452	0.0452	0.0409	0.0415	0.0416
***N***	10597	10597	10597	10597	10597	10597	13816	13816	13816	13816	13816	13816

Notes: For each variable, we report the regression coefficient and t-statistic (in parentheses) calculated

***,**, and * represent 1%, 5%, and 10% significant levels.

Table 7 presents the regression results of a split sample of different industries based on Eqs (1)–(6). The regression results of columns (1) through (12) indicate that the coefficients of IFPLEDGE_t_ in samples of manufacturing industry are negative and statistically significant at the 1%, 5% and 10% levels, respectively. And the interaction coefficients of IFPLEDGE_t_ and SP_t_ (IFPLEDGE_t__SP_t_) and the interaction coefficients of IFPLEDGE_t_ and IP_t_ (IFPLEDGE_t__IP_t_) are negative and significant at the 1% and 5% levels, whereas the coefficients in the sample of technology industry are less significant, indicating that the effect of reducing stock price crash risk is more pronounced in the sample of manufacturing industry in the case of equity pledge through insurance participation. This is mainly because manufacturing companies usually have more stable share prices due to their stable earnings and cash flow performance, as well as investors’ preference for their solid dividend income, and in the case of equity pledges, the risk-reducing effect of insurer participation on the risk of their share prices crashing is more pronounced. In contrast, technology companies, due to their innovative and high growth potential, may show higher share price increases when the market expects future growth, but also face greater market volatility risk, which is not obvious in the case of equity pledges, even if there is the behavior of insurers’ participation in the reduction of the risk of a collapse of their share prices.

**Table 7:** Test results of industry classification.

	Sample of manufacturing industry						Sample of technology industry					
	NCSKEW _t+1_			DUVOL _t+1_			NCSKEW _t+1_			DUVOL _t+1_		
	(1)	(2)	(3)	(4)	(5)	(6)	(7)	(8)	(9)	(10)	(11)	(12)
**IFPLEDGE**_**t**_	-0.0221**	-0.0832**	-0.1011***	-0.0851**	-0.0403*	-0.0513*	-0.0091	-0.0092	-0.0081	-0.0022	-0.0012	0.0401
	(0.0353)	(0.0151)	(0.0082)	(0.0192)	(0.0904)	(0.0583)	(0.4701)	(0.4152)	(0.2992)	(0.8371)	(0.9123)	(0.1121)
**IFPLEDGE**_**t**_ **_IP**_**t**_		-0.0491**			-0.0354**			-0.0693			-0.0412	
		(0.0201)			(0.0183)			(0.3123)			(0.4001)	
**IFPLEDGE**_**t**_ **_SP**_**t**_			-6.6322***			-4.5304***			-1.3301			0.0832
			(0.0021)			(0.0033)			(0.8413)			(0.9861)
**IP**_**t**_		-0.0041			-0.0044			0.0082			0.0212	
		(0.8182)			(0.7722)			(0.8961)			(0.6142)	
**SP**_**t**_			-1.0442			-0.2182			-0.8501			-0.8341
			(0.5432)			(0.8581)			(0.8743)			(0.8253)
**TOPHLD**_**t**_	-0.0371	-0.0391	-0.0343	-0.0372	-0.0383	-0.0343	-0.0681	-0.0681	-0.0701	-0.0771	-0.0784	-0.0792
	(0.2892)	(0.2716)	(0.3281)	(0.1382)	(0.1252)	(0.1702)	(0.5691)	(0.5712)	(0.5572)	(0.3624)	(0.3513)	(0.3493)
**DTA**_**t**_	-0.0581*	-0.0535*	-0.0543*	-0.0381*	-0.0343	-0.0355*	0.0601	0.0532	0.0593	0.0662	0.0622	0.0652
	(0.0534)	(0.0762)	(0.0712)	(0.0692)	(0.1083)	(0.0994)	(0.5072)	(0.5564)	(0.5144)	(0.3013)	(0.3325)	(0.3035)
**SOE**_**t**_	-0.0392***	-0.0391***	-0.0391***	-0.0271***	-0.0264***	-0.0263***	-0.1203***	-0.1193***	-0.1193***	-0.0472	-0.0473	-0.0462
	(0.0013)	(0.0020)	(0.0022)	(0.0022)	(0.0023)	(0.0023)	(0.0082)	(0.0092)	(0.0082)	(0.1343)	(0.1345)	(0.1425)
**MTB**_**t**_	0.03431***	0.0331***	0.0331***	0.0181***	0.0162***	0.0161***	0.0211	0.0232	0.0223	0.0051	0.0063	0.0062
	(0.0001)	(0.0001)	(0.0001)	(0.00002)	(0.0001)	(0.0001)	(0.1851)	(0.1422)	(0.1701)	(0.6353)	(0.6162)	(0.6172)
**ROA**_**t**_	0.5261***	0.5122***	0.5153***	0.2932***	0.2794***	0.2824***	0.5702*	0.5811*	0.5753*	0.3315	0.3321	0.3341
	(0.0000)	(0.0000)	(0.0000)	(0.0001)	(0.0002)	(0.0002)	(0.0741)	(0.0682)	(0.0714)	(0.1392)	(0.1401)	(0.1362)
**SIZE**_**t**_	-0.0062	-0.0112	-0.0112	-0.0213***	-0.0262***	-0.0253***	-0.0372*	-0.0315	-0.0351*	-0.0512***	-0.0502***	-0.0501***
	(0.3673)	(0.1362)	(0.1461)	(0.0003)	(0.0000)	(0.0000)	(0.0832)	(0.1482)	(0.0971)	(0.0010)	(0.0010)	(0.0010)
**DTURN**_**t**_	-5.5182***	-5.4871***	-5.5202***	-4.0463***	-4.0105***	-4.0406***	-8.3511***	-8.3252***	-8.3244***	-5.9123***	-5.9091***	-5.9032***
	(0.0000)	(0.0000)	(0.0000)	(0.0000)	(0.0000)	(0.0000)	(0.0000)	(0.0000)	(0.0000)	(0.0000)	(0.0000)	(0.0000)
**ABACC**_**t**_	0.2291	0.2523	0.2535	0.1622	0.1843	0.1852	0.7312	0.6573	0.7351	0.8183	0.7823	0.8191
	(0.3871)	(0.3412)	(0.3392)	(0.3841)	(0.3242)	(0.3224)	(0.3825)	(0.4324)	(0.3792)	(0.1645)	(0.1852)	(0.1644)
**RET**_**t**_	8.7272***	8.7701***	8.7723***	6.0991***	6.1382***	6.1464***	5.4964***	5.4072***	5.4385***	3.9722***	3.9663***	3.9626***
	(0.0001)	(0.0001)	(0.0001)	(0.0000)	(0.0000)	(0.0000)	(0.0020)	(0.0020)	(0.0020)	(0.0010)	(0.0010)	(0.0010)
**SIGMA**_**t**_	-3.440***	-3.435***	-3.421***	-2.657***	-2.648***	-2.635***	-3.606***	-3.559***	-3.603***	-2.954***	-2.945***	-2.959***
	(0.0000)	(0.0000)	(0.0000)	(0.0000)	(0.0000)	(0.0000)	(0.0000)	(0.0000)	(0.0000)	(0.0000)	(0.0000)	(0.0000)
**CP**_**t**_	0.021**	0.021**	0.021**	0.011	0.011	0.011	0.011	0.009	0.01	-0.001	-0.001	-0.001
	(0.0451)	(0.0426)	(0.0435)	(0.1276)	(0.1215)	(0.1223)	(0.7066)	(0.7523)	(0.7293)	(0.9671)	(0.9662)	(0.9534)
**IDR**_**t**_	0.003	0.001	0.0003	-0.002	-0.004	-0.005	-0.237	-0.24	-0.241	-0.037	-0.036	-0.037
	(0.9782)	(0.9954)	(0.9983)	(0.9715)	(0.9486)	(0.9412)	(0.3901)	(0.3832)	(0.3824)	(0.8513)	(0.8554)	(0.8492)
**SVS**_**t**_	-0.0021	-0.0021	-0.0021	-0.0012	-0.0011	-0.0012	0.0301	0.0291	0.0302	0.0142	0.0141	0.0151
	(0.6612)	(0.6821)	(0.6551)	(0.7912)	(0.8162)	(0.7851)	(0.1241)	(0.1292)	(0.1211)	(0.2882)	(0.3100)	(0.2801)
**NCSKEW**_**t**_	0.0491***	0.0492***	0.0494***				0.0213	0.0212	0.0211			
	(0.0001)	(0.0001)	(0.0001)				(0.3601)	(0.3532)	(0.3591)			
**DUVOL**_**t**_				0.0441***	0.0443***	0.0432***				0.0193	0.0194	0.0191
				(0.0001)	(0.0001)	(0.0001)				(0.4101)	(0.4152)	(0.4091)
**_cons**	34.3374***	33.0762***	33.1603***	21.1991***	20.0321***	20.0972***	58.8984***	60.3533***	59.5032***	34.9501***	35.2193***	35.2321***
	(0.0000)	(0.0000)	(0.0000)	(0.0000)	(0.0000)	(0.0000)	(0.0000)	(0.0000)	(0.0000)	(0.0000)	(0.0000)	(0.0000)
**Industry**	YES	YES	YES	YES	YES	YES	YES	YES	YES	YES	YES	YES
**Year**	YES	YES	YES	YES	YES	YES	YES	YES	YES	YES	YES	YES
**Firm**	YES	YES	YES	YES	YES	YES	YES	YES	YES	YES	YES	YES
***R*** ^**2**^	0.047	0.0452	0.0463	0.044	0.0459	0.0404	0.042	0.0416	0.0412	0.041	0.042	0.0400
***N***	18384	18384	18384	18384	18384	18384	2,004	2,004	2,004	2,004	2,004	2,004

Notes: For each variable, we report the regression coefficient and t-statistic (in parentheses) calculated

***,**, and * represent 1%, 5%, and 10% significant levels.

### 4.4 Endogenous test

Primarily endogenous sources include reverse causality (also known as simultaneity bias), missing variables, and measurement of errors [49]. In consideration of reverse causality, this paper utilizes the first-order lag term of response variables to mitigate a potential endogenous issue.

In the robustness testing, Tables 10 and 11 of the lagged second period of the explanatory variables and the three lags of the explanatory variables are also consistent with the available results. In addition, Table 10 was subjected to reverse causality tests, which further examined the issue of reverse causality. Regarding missing variables, we may have omitted unobservable variables such as risk appetite, personal financial knowledge, and risk prevention abilities. These variables may influence crash risk indirectly. Despite the reality that measurement errors are always present in social survey data, this paper concludes that the impact on endogenous is minimal due to the strict quality control of the survey data used in this study.

The results of GMM test are shown in Table 8. The results show that there is first-order autocorrelation in the difference of the perturbation terms, but not second-order autocorrelation, so the original hypothesis of no autocorrelation in the perturbation terms is accepted; the corresponding P-values of Sargan’s test are all greater than 0.5, which indicates that the regression results are not over-identified. The coefficients of the equity pledge (IFPLEDGE_t_) in columns (1) through (4) are negative and statistically significant at the 1%,5% and 10% level, respectively. And there is a negative and significant relationship between the interaction of IFPLEDGE_t_ and SPt (IFPLEDGE_t__SP_t_) and the interaction of IFPLEDGE_t_ and IP_t_ (IFPLEDGE_t__IP_t_) and stock price crash risk, respectively; H2A is further validated.

**Table 8:** Results of GMM test.

	NCSKEW _t+1_		DUVOL _t+1_	
	(1)	(2)	(3)	(4)
**IFPLEDGE**_**t**_	-0.3587***	-0.2634***	-0.1645**	-0.0993*
	(3.0038)	(3.0451)	(2.1285)	(0.0165)
**IFPLEDGE**_**t**_ **_IP**_**t**_	-1.0606***		-0.5928***	
	(-3.2707)		(-2.8397)	
**IFPLEDGE**_**t**_ **_SP**_**t**_		-0.4458***		-1.2265**
		(-3.6208)		(-1.4556)
**IP**_**t**_	-0.6052***		-0.3090**	
	(2.7219)		(2.1639)	
**SP**_**t**_		-0.2830***		-1.9462*
		(3.3661)		(0.3095)
**TOPHLD**_**t**_	-0.8786	-4.6054***	-0.6958	-0.7598
	(-0.9486)	(-4.5137)	(-1.1676)	(-1.2658)
**DTA**_**t**_	-0.1018	-0.3506	0.2837	0.4331
	(-0.1515)	(-0.7323)	(0.6339)	(0.9822)
**SOE**_**t**_	0.4323*	-1.4158***	0.1630	0.1217
	(1.6786)	(-3.2683)	(0.9677)	(0.7623)
**MTB**_**t**_	0.2510***	0.0894***	0.1656***	0.1520***
	(6.8179)	(3.3760)	(6.6598)	(6.6035)
**ROA**_**t**_	1.8023	11.2305***	0.9519	1.2620
	(1.0468)	(9.5249)	(0.8059)	(1.0678)
**SIZE**_**t**_	-0.2921***	-0.2586***	-0.2333***	-0.2212***
	(-3.2533)	(-3.2339)	(-3.9356)	(-3.7394)
**DTURN**_**t**_	-4.7481***	-2.0905	-6.4943***	-6.8388***
	(-2.6717)	(-1.3679)	(-5.3903)	(-5.7372)
**ABACC**_**t**_	7.2814*	5.9956**	4.2800	2.9117
	(1.8582)	(2.4843)	(1.5754)	(1.0701)
**RET**_**t**_	-4.4025***	3.2168***	-3.3544***	-3.0844***
	(-2.9219)	(2.8646)	(-3.2940)	(-3.1529)
**SIGMA**_**t**_	-5.8161***	-3.0467***	-4.9705***	-5.0306***
	(-9.1627)	(-6.0014)	(-11.7611)	(-11.9602)
**CP**_**t**_	0.6936*	-0.1219	0.3866	0.2378
	(1.6717)	(-0.5465)	(1.3938)	(0.9146)
**IDR**_**t**_	-8.9547***	-7.4136***	-4.8660**	-3.7653*
	(-2.8300)	(-4.0156)	(-2.3292)	(-1.8312)
**SVS**_**t**_	-0.0817	0.1212	-0.0604	-0.0662
	(-0.5629)	(0.8179)	(-0.6327)	(-0.6955)
**NCSKEW**_**t**_	-0.2943***	-0.4637***	-0.3009***	-0.2981***
	(-31.9564)	(-50.1298)	(-29.6048)	(-29.6362)
**DUVOL**_**t**_	9.0488***	-4.6054***	6.6002***	6.0495***
	(4.2929)	(-4.5137)	(4.5161)	(4.1340)
**_cons**	-0.8786	-0.3506	-0.6958	-0.7598
	(-0.9486)	(-0.7323)	(-1.1676)	(-1.2658)
**Industry**	YES	YES	YES	YES
**Year**	YES	YES	YES	YES
**Firm**	YES	YES	YES	YES
***N***	27793	27793	27793	27793
**AR(1)**	0.013	0.016	0.014	0.015
**AR(2)**	0.471	0.742	0.731	0.523
**Sargan test**	0.51	0.84	0.77	0.54

Notes: For each variable, we report the regression coefficient and t-statistic (in parentheses) calculated

***,**, and * represent 1%, 5%, and 10% significant levels.

Simultaneously, the robustness analysis section of this paper uses PSM analysis to address the problem prompted by sample selection deviation in order to weaken the endogenous problem further.

### 4.5 Robustness test and additional analysis

#### 4.5.1 PSM test

Propensity Score Matching (PSM) is a statistical method used to process data from Observational Studies. In Observational Study, due to various reasons, there are more data bias and confounding variable, the method of Propensity Score Matching (PSM) is designed to reduce the effect of these bias and confounding variable in order to make a more reasonable comparison between the experimental group and the control group. In order to prevent endogenous problems caused by the systematic difference between the pledge company and the non-pledge company, this paper employs the PSM approach to identify the pairing sample of the company of equity pledge leveraging the matching tendency score (Propensity Score, abbreviated PS) and tests the relationship between insurance participation, equity pledge, and stock price crash risk. We choose thirteen corporate characteristic variables to establish a tendentious model about equity pledge, such as the first largest shareholder(TOPHLD), the property nature(SOE), the company’s market to book value ratio(MTB), the asset-liability ratio(DTA), the asset yield(ROA), the asset size(SIZE) and the detrended monthly share turnover(DTURN), the absolute value of discretionary accruals(ABACC), firm-specific weekly stock Returns(RET), firm-specific week stock returns(SIGMA), whether the general manager and the chairman of the board are one person (CP), independent directors to the size of the board of directors (IDR), board size(SVS), use Logit model to estimate the tendentious score value (PS value) of each company’s equity pledge, choose the company of no stock and get the closest PS value as a pairing sample in the same year for each company with equity pledge, and examine the differences of variables between the two groups of samples.

The results of the PSM pairing sample robustness test are presented in Table 9. The coefficients of equity pledge (IFPLEDGE_t_) in columns (1) through (6) are significant and negative. The interaction of IFPLEDGEt and SP_t_ (IFPLEDGE_t__SP_t_) and the interaction of IFPLEDGE_t_ and IP_t_ (IFPLEDGE_t__IP_t_) are negative and significant. Hypothesis H2A is further supported.

**Table 9:** The robustness test of PSM.

	NCSKEW _t+1_			DUVOL _t+1_		
	(1)	(2)	(3)	(4)	(5)	(6)
**IFPLEDGE**_**t**_	-0.0175*	-0.0242*	-0.0255*	-0.0230**	-0.0293***	-0.0286***
	(0.0095)	(0.0131)	(0.0147)	(0.0095)	(0.0105)	(0.0101)
**IFPLEDGE**_**t**_ **_IP**_**t**_		-0.0240*			0.0223*	
		(0.0129)			(0.0121)	
**IFPLEDGE**_**t**_ **_SP**_**t**_			-3.8719*			-2.6785*
			(2.0933)			(1.4223)
**IP**_**t**_		-0.0046*			-0.0019*	
		(0.0002)			(0.0001)	
**SP**_**t**_			-0.7086*			-0.0686
			(0.0038)			(1.1382)
**TOPHLD**_**t**_	-0.0370	-0.0399	-0.0349	-0.0380	-0.0404	-0.0359
	(0.0836)	(0.0837)	(0.0836)	(0.0576)	(0.0577)	(0.0577)
**DTA**_**t**_	-0.1704***	-0.1637***	-0.1640***	-0.0849**	-0.0792**	-0.0792**
	(0.0497)	(0.0498)	(0.0497)	(0.0340)	(0.0340)	(0.0339)
**SOE**_**t**_	-0.0239	-0.0236	-0.0235	-0.0184	-0.0182	-0.0179
	(0.0358)	(0.0358)	(0.0358)	(0.0235)	(0.0235)	(0.0234)
**MTB**_**t**_	0.0778***	0.0766***	0.0770***	0.0530***	0.0520***	0.0522***
	(0.0076)	(0.0076)	(0.0076)	(0.0052)	(0.0052)	(0.0052)
**ROA**_**t**_	0.3488***	0.3486***	0.3508***	0.2090**	0.2090**	0.2106**
	(0.1324)	(0.1324)	(0.1325)	(0.0915)	(0.0915)	(0.0915)
**SIZE**_**t**_	0.0418***	0.0377***	0.0390***	0.0126	0.0093	0.0099
	(0.0132)	(0.0133)	(0.0133)	(0.0091)	(0.0092)	(0.0092)
**DTURN**_**t**_	-5.8414***	-5.8194***	-5.8455***	-4.3305***	-4.3127***	-4.3354***
	(0.5050)	(0.5050)	(0.5050)	(0.3488)	(0.3488)	(0.3488)
**ABACC**_**t**_	0.3868	0.3969	0.3956	0.2978*	0.3064*	0.3055*
	(0.2519)	(0.2518)	(0.2518)	(0.1729)	(0.1729)	(0.1729)
**RET**_**t**_	5.1174***	5.1428***	5.1479***	3.4063***	3.4258***	3.4421***
	(0.5325)	(0.5320)	(0.5327)	(0.3728)	(0.3724)	(0.3729)
**SIGMA**_**t**_	-3.4732***	-3.4677***	-3.4704***	-2.7679***	-2.7638***	-2.7634***
	(0.2372)	(0.2373)	(0.2372)	(0.1661)	(0.1662)	(0.1661)
**CP**_**t**_	0.0220	0.0220	0.0219	0.0049	0.0049	0.0049
	(0.0159)	(0.0159)	(0.0159)	(0.0108)	(0.0108)	(0.0108)
**IDR**_**t**_	-0.0530	-0.0571	-0.0558	-0.0262	-0.0294	-0.0294
	(0.1467)	(0.1467)	(0.1468)	(0.1016)	(0.1015)	(0.1016)
**SVS**_**t**_	-0.0194*	-0.0192*	-0.0192*	-0.0137*	-0.0136*	-0.0135*
	(0.0104)	(0.0103)	(0.0103)	(0.0076)	(0.0075)	(0.0075)
**NCSKEW**_**t**_	-0.0973***	-0.0974***	-0.0976***			
	(0.0071)	(0.0070)	(0.0071)			
**DUVOL**_**t**_				-0.0604***	-0.0605***	-0.0607***
				(0.0049)	(0.0049)	(0.0049)
**_cons**	41.5294***	40.3321***	40.7542***	22.7844***	21.8148***	22.0235***
	(4.2627)	(4.3039)	(4.2771)	(2.9044)	(2.9355)	(2.9176)
**Industry**	YES	YES	YES	YES	YES	YES
**Year**	YES	YES	YES	YES	YES	YES
**Firm**	YES	YES	YES	YES	YES	YES
***R*** ^**2**^	0.0407	0.0409	0.0409	0.0374	0.0377	0.0378
***N***	24398	24398	24398	24398	24398	24398

Notes: For each variable, we report the regression coefficient and t-statistic (in parentheses) calculated

***,**, and * represent 1%, 5%, and 10% significant levels.

#### 4.5.2 Test results for explanatory variables lagged by two and three periods

Tables 10 and 11 examine the explanatory variables with two and three lags, and we discovered that the coefficients of equity pledge (IFPLEDGE_t_) from column (1) to column (6) are negative and statistically significant. Consistent with previous research, both the interaction of IFPLEDGE_t_ and SP_t_ (IFPLEDGE_t__SP_t_) and the interaction of IFPLEDGE_t_ and IP_t_ (IFPLEDGE_t__IP_t_) are negative and statistically significant.

**Table 10:** Test results of the explanatory variables with two lags.

	NCSKEW2 _t+1_			DUVOL2 _t+1_		
	(1)	(2)	(3)	(4)	(5)	(6)
**IFPLEDGE**_**t**_	-0.0178*	-0.0247*	-0.0260*	-0.0233**	-0.0297***	-0.0288***
	(0.0094)	(0.0130)	(0.0147)	(0.0095)	(0.0105)	(0.0101)
**IFPLEDGE**_**t**_ **_IP**_**t**_		-0.0248*			-0.0228*	
		(0.0127)			(0.0117)	
**IFPLEDGE**_**t**_ **_SP**_**t**_			-3.9506*			-2.6947*
			(2.0897)			(1.4209)
**IP**_**t**_		-0.0040*			-0.0015*	
		(0.0021)			(0.0008)	
**SP**_**t**_			-0.8656*			-0.0038
			(0.4558)			(1.1365)
**TOPHLD**_**t**_	-0.0456	-0.0484	-0.0436	-0.0431	-0.0456	-0.0411
	(0.0836)	(0.0837)	(0.0837)	(0.0576)	(0.0577)	(0.0577)
**DTA**_**t**_	-0.1651***	-0.1584***	-0.1588***	-0.0817**	-0.0760**	-0.0760**
	(0.0499)	(0.0500)	(0.0499)	(0.0341)	(0.0341)	(0.0341)
**SOE**_**t**_	-0.0245	-0.0242	-0.0242	-0.0188	-0.0186	-0.0183
	(0.0358)	(0.0358)	(0.0357)	(0.0234)	(0.0235)	(0.0234)
**MTB**_**t**_	0.0776***	0.0764***	0.0769***	0.0528***	0.0519***	0.0520***
	(0.0076)	(0.0076)	(0.0076)	(0.0052)	(0.0052)	(0.0052)
**ROA**_**t**_	0.3453***	0.3454***	0.3472***	0.2080**	0.2082**	0.2096**
	(0.1324)	(0.1324)	(0.1325)	(0.0915)	(0.0915)	(0.0915)
**SIZE**_**t**_	0.0418***	0.0378***	0.0392***	0.0127	0.0094	0.0101
	(0.0132)	(0.0133)	(0.0133)	(0.0091)	(0.0092)	(0.0092)
**DTURN**_**t**_	-5.8103***	-5.7886***	-5.8137***	-4.3044***	-4.2867***	-4.3089***
	(0.5047)	(0.5046)	(0.5046)	(0.3486)	(0.3485)	(0.3486)
**ABACC**_**t**_	0.4113	0.4215*	0.4202*	0.3132*	0.3218*	0.3210*
	(0.2520)	(0.2519)	(0.2520)	(0.1730)	(0.1729)	(0.1729)
**RET**_**t**_	5.0768***	5.1022***	5.1042***	3.3843***	3.4039***	3.4186***
	(0.5320)	(0.5315)	(0.5322)	(0.3723)	(0.3720)	(0.3725)
**SIGMA**_**t**_	-3.4483***	-3.4430***	-3.4461***	-2.7528***	-2.7489***	-2.7486***
	(0.2371)	(0.2372)	(0.2371)	(0.1661)	(0.1662)	(0.1661)
**CP**_**t**_	0.0231	0.0231	0.0230	0.0058	0.0058	0.0057
	(0.0160)	(0.0159)	(0.0159)	(0.0108)	(0.0108)	(0.0108)
**IDR**_**t**_	-0.0400	-0.0441	-0.0425	-0.0173	-0.0206	-0.0204
	(0.1466)	(0.1465)	(0.1466)	(0.1014)	(0.1014)	(0.1014)
**SVS**_**t**_	-0.0201*	-0.0199*	-0.0199*	-0.0141*	-0.0139*	-0.0138*
	(0.0104)	(0.0103)	(0.0104)	(0.0076)	(0.0075)	(0.0075)
**NCSKEW**_**t**_	-0.0967***	-0.0968***	-0.0970***			
	(0.0071)	(0.0070)	(0.0071)			
**DUVOL**_**t**_				-0.0602***	-0.0603***	-0.0604***
				(0.0049)	(0.0049)	(0.0049)
**_cons**	41.5072***	40.3165***	40.7705***	22.7722***	21.8056***	22.0307***
	(4.2644)	(4.3057)	(4.2787)	(2.9027)	(2.9342)	(2.9159)
**Industry**	YES	YES	YES	YES	YES	YES
**Year**	YES	YES	YES	YES	YES	YES
**Firm**	YES	YES	YES	YES	YES	YES
***R*** ^**2**^	0.0402	0.0404	0.0405	0.0370	0.0374	0.0374
***N***	23413	23413	23413	23413	23413	23413

Notes: For each variable, we report the regression coefficient and t-statistic (in parentheses) calculated

***,**, and * represent 1%, 5%, and 10% significant levels.

**Table 11:** Test results for explanatory variables lagged three periods.

	NCSKEW3 _t+1_			DUVOL3 _t+1_		
	(1)	(2)	(3)	(4)	(5)	(6)
**IFPLEDGE**_**t**_	-0.014*	-0.011*	-0.012*	-0.009*	-0.007	-0.008*
	(0.007)	(0.005)	(0.006)	(0.004)	(0.391)	(0.004)
**IFPLEDGE**_**t**_ **_IP**_**t**_		-0.009**			-0.007*	
		(0.003)			(0.003)	
**IFPLEDGE**_**t**_ **_SP**_**t**_			-0.882*			-0.007
			(0.404)			(0.594)
**IP**_**t**_		-0.01			-0.004*	
		(0.530)			(0.002)	
**SP**_**t**_			-0.438*			-0.031*
			(0.214)			(0.015)
**TOPHLD**_**t**_	-0.006	-0.006	-0.008	0.012**	0.012**	0.011**
	(0.854)	(0.844)	(0.818)	(0.005)	(0.004)	(0.004)
**DTA**_**t**_	-0.023*	-0.022*	-0.024*	-0.024	-0.024	-0.024
	(0.011)	(0.012)	(0.012)	(0.231)	(0.231)	(0.222)
**SOE**_**t**_	0.008	0.007	0.008	-0.002	-0.002	-0.002
	(0.489)	(0.504)	(0.472)	(0.796)	(0.788)	(0.807)
**MTB**_**t**_	0.011**	0.011*	0.012**	0.011***	0.011***	0.011***
	(0.044)	(0.052)	(0.037)	(0.007)	(0.007)	(0.006)
**ROA**_**t**_	-0.158	-0.161	-0.153	-0.179**	-0.179**	-0.176**
	(0.123)	(0.117)	(0.137)	(0.014)	(0.014)	(0.015)
**SIZE**_**t**_	0.01	0.01	0.012*	0.010**	0.010**	0.011**
	(0.119)	(0.157)	(0.086)	(0.027)	(0.032)	(0.021)
**DTURN**_**t**_	0.083	0.09	0.085	0.166*	0.167*	0.167*
	(0.885)	(0.877)	(0.884)	(0.082)	(0.082)	(0.081)
**ABACC**_**t**_	0.03	0.031	0.025	-0.103	-0.104	-0.106
	(0.899)	(0.898)	(0.918)	(0.539)	(0.534)	(0.528)
**RET**_**t**_	0.682	0.692	0.664	0.295	0.298	0.288
	(0.176)	(0.171)	(0.188)	(0.407)	(0.402)	(0.418)
**SIGMA**_**t**_	0.189*	0.195*	0.183*	0.113	0.114	0.11
	(0.097)	(0.094)	(0.095)	(0.484)	(0.477)	(0.495)
**CP**_**t**_	0.009*	0.009*	0.009*	0.002	0.002	0.002
	(0.004)	(0.004)	(0.004)	(0.785)	(0.789)	(0.789)
**IDR**_**t**_	-0.054**	-0.056**	-0.054**	0.004	0.004	0.004
	(0.015)	(0.015)	(0.015)	(0.952)	(0.956)	(0.948)
**SVS**_**t**_	-0.005*	-0.005*	-0.006	-0.004	-0.004	-0.004
	(0.099)	(0.091)	(0.194)	(0.168)	(0.168)	(0.165)
**NCSKEW**_**t**_	-0.006	-0.006	-0.006			
	(0.374)	(0.370)	(0.388)			
**DUVOL**_**t**_				-0.015**	-0.015**	-0.015**
				(0.037)	(0.037)	(0.038)
**Constant**	-0.745	-0.843	-0.529	0.863	0.866	0.968
	(0.815)	(0.791)	(0.868)	(0.699)	(0.699)	(0.665)
**Industry**	YES	YES	YES	YES	YES	YES
**Year**	YES	YES	YES	YES	YES	YES
**Firm**	YES	YES	YES	YES	YES	YES
**R** ^**2**^	0.014	0.014	0.014	0.015	0.015	0.015
**N**	22,003	22,003	22,003	22,003	22,003	22,003

Notes: For each variable, we report the regression coefficient and t-statistic (in parentheses) calculated

***,**, and * represent 1%, 5%, and 10% significant levels.

#### 4.5.3 Reverse causality issues

Strong evidence that firms with equity pledges have a lower risk of collapse in the future was found in our earlier findings. However, it is important to note that firms with a lower risk of future collapse are more likely to make equity pledges than firms with a higher risk of future collapse. In this instance, causality can therefore shift in the opposite direction. To further insulate our results from the problem of reverse causality, we employ the change and reverse change tests of Aggarwal et al. [50]. This approach also mitigates the impact of unobservable, time-invariant firm characteristics. We assume that, if a firm’s equity pledges affect future crash risk, the future crash risk will decrease correspondingly as the firm’s equity pledges increase.

Panel A Table 12 displays the outcomes of the change tests. The dependent variables (ΔNCSKEW_t+1_ and ΔDUVOL_t+1_) refer to the change from t-period to t+1. Our major explanatory variables (ΔIFPLEDGE_t_, ΔIFPLEDGE_t_*ΔIP_t_, and ΔIFPLEDGE_t_*ΔSP_t_) and control variables refer to the change from period t-1 to period t. As expected, we observed significantly negative coefficients for ΔIFPLEDGE_t_, ΔIFPLEDGE_t_*ΔIP_t_, and ΔIFPLEDGE_t_*ΔSP_t_, which supported our primary prediction. To investigate the issue of reverse causality further, we conduct a reverse change regression using the change in equity pledges and the change in insurance participation as dependent variables.

**Table 12:** Robustness check using change and reverse change tests. Panel A. Change tests; Panel B. Reverse change tests.

Variables	△NCSKEW_t+1_			△DUVOL _t+1_		
	(1)	(2)	(3)	(4)	(5)	(6)
**△IFPLEDGE**_**t**_	-0.025*	-0.023*	-0.025*	-0.019**	-0.011	-0.019**
	(0.057)	(0.055)	(0.055)	(0.044)	(0.299)	(0.041)
**△IFPLEDGE**_**t**_ **_△IP**_**t**_		-0.019*			-0.017*	
		(0.047)			(0.032)	
**△IFPLEDGE**_**t**_ **_△SP**_**t**_			-3.833**			-3.036**
			(0.043)			(0.023)
**△IP**_**t**_		-0.014*			-0.003*	
		(0.036)			(0.006)	
**△SP**_**t**_			-0.025			-0.001
			(0.054)			(0.998)
**△TOPHLD**_**t**_	-0.164	-0.161	-0.163	-0.144*	-0.145*	-0.143*
	(0.145)	(0.152)	(0.147)	(0.069)	(0.068)	(0.070)
**△DTA**_**t**_	-0.154**	-0.153**	-0.148**	-0.072*	-0.071	-0.068
	(0.013)	(0.014)	(0.017)	(0.098)	(0.102)	(0.120)
**△SOE**_**t**_	0.013	0.013	0.012	0.018	0.018	0.017
	(0.752)	(0.741)	(0.762)	(0.528)	(0.530)	(0.537)
**△MTB**_**t**_	0.111***	0.111***	0.111***	0.077***	0.077***	0.077***
	(0.000)	(0.000)	(0.000)	(0.000)	(0.000)	(0.000)
**△ROA**_**t**_	0.236*	0.232*	0.237*	0.205**	0.203**	0.206**
	(0.069)	(0.074)	(0.068)	(0.025)	(0.026)	(0.024)
**△SIZE**_**t**_	0.140***	0.139***	0.138***	0.097***	0.097***	0.095***
	(0.000)	(0.000)	(0.000)	(0.000)	(0.000)	(0.000)
**△DTURN**_**t**_	-0.047	-0.049	-0.047	-0.031	-0.034	-0.032
	(0.244)	(0.221)	(0.239)	(0.267)	(0.222)	(0.259)
**△ABACC**_**t**_	-0.035	-0.037	-0.035	-0.075	-0.075	-0.075
	(0.857)	(0.849)	(0.855)	(0.580)	(0.576)	(0.578)
**△RET**_**t**_	1.160***	1.164***	1.177***	0.655**	0.660**	0.668**
	(0.008)	(0.008)	(0.008)	(0.034)	(0.033)	(0.030)
**△SIGMA**_**t**_	-2.734***	-2.732***	-2.738***	-2.152***	-2.149***	-2.156***
	(0.000)	(0.000)	(0.000)	(0.000)	(0.000)	(0.000)
**△CP**_**t**_	0.007	0.008	0.007	-0.002	-0.002	-0.002
	(0.679)	(0.676)	(0.682)	(0.884)	(0.882)	(0.879)
**△IDR**_**t**_	-0.293*	-0.294*	-0.296*	-0.165	-0.162	-0.167
	(0.057)	(0.056)	(0.054)	(0.127)	(0.133)	(0.122)
**△SVS**_**t**_	-0.005	-0.005	-0.005	0.001	0.001	0.001
	(0.664)	(0.668)	(0.662)	(0.926)	(0.926)	(0.928)
**△NCSKEW**_**t**_	-0.491***	-0.491***	-0.491***			
	(0.000)	(0.000)	(0.000)			
**△DUVOL**_**t**_				-0.493***	-0.493***	-0.493***
				(0.000)	(0.000)	(0.000)
**Constant**	-33.729***	-33.701***	-33.970***	-27.169***	-27.376***	-27.359***
	(0.000)	(0.000)	(0.000)	(0.000)	(0.000)	(0.000)
**Industry**	Yes	Yes	Yes	Yes	Yes	Yes
**Year**	Yes	Yes	Yes	Yes	Yes	Yes
**Firm**	Yes	Yes	Yes	Yes	Yes	Yes
**R** ^**2**^	0.256	0.256	0.256	0.264	0.264	0.264
**N**	21,472	21,472	21,472	21,472	21,472	21,472
**Variables**	△IFPLEDGE_t_	△IFPLEDGE_t__△IP_t_	△IFPLEDGE_t__△SP_t_	△IFPLEDGE_t_	△IFPLEDGE_t__△IP_t_	△IFPLEDGE_t__△SP_t_
	(1)	(2)	(3)	(4)	(5)	(6)
**△NCSKEW**_**t+1**_	0.007	0.002	-0.0001			
	(0.087)	(0.327)	(0.093)			
**△DUVOL**_**t+1**_				0.010	0.004	-0.0001
				(0.094)	(0.212)	(0.123)
**△IFPLEDGE**_**t**_		0.251	0.0001		0.251	0.0001
		(0.100)	(0.107)		(0.100)	(0.107)
**△IP**_**t**_		-0.450			-0.450	
		(0.100)			(0.200)	
**△SP**_**t**_			-0.029			-0.029
			(0.102)			(0.200)
**△TOPHLD**_**t**_	-0.099***	-0.049	0.0003	-0.099***	-0.049	0.0002
	(0.00001)	(0.208)	(0.538)	(0.00001)	(0.209)	(0.543)
**△DTA**_**t**_	0.003	-0.044**	0.001***	0.003	-0.044**	0.001***
	(0.551)	(0.042)	(0.000)	(0.555)	(0.041)	(0.000)
**△SOE**_**t**_	0.067	-0.012	-0.0001	0.067	-0.012	-0.0001
	(0.325	(0.382	(0.398	(0.325)	(0.381)	(0.401)
**△MTB**_**t**_	-0.015	0.005*	-0.0001***	-0.015	0.005*	-0.0001***
	(0.103)	(0.068)	(0.0002)	(0.104)	(0.069)	(0.0002)
**△ROA**_**t**_	0.765***	0.160***	0.0002	0.765***	0.159***	0.0002
	(0.000)	(0.0004)	(0.749)	(0.000)	(0.0004)	(0.740)
**△SIZE**_**t**_	-0.027	0.007	-0.0005***	-0.025	0.007	-0.0005***
	(0.787)	(0.238)	(0.000)	(0.801)	(0.239)	(0.000)
**△DTURN**_**t**_	0.541**	0.158***	-0.0004***	0.573**	0.158***	-0.0004***
	(0.018)	(0.000)	(0.010)	(0.013)	(0.000)	(0.010)
**△ABACC**_**t**_	0.313***	0.069	-0.0001	0.320***	0.069	-0.0001
	(0.003)	(0.302)	(0.844)	(0.003)	(0.298)	(0.840)
**△RET**_**t**_	0.005	-0.260*	0.004***	0.005	-0.256*	0.005***
	(0.584)	(0.086)	(0.005)	(0.587)	(0.092)	(0.005)
**△SIGMA**_**t**_	0.098	-0.135*	-0.001*	0.097	-0.133*	-0.001*
	(0.225)	(0.052)	(0.085)	(0.230)	(0.057)	(0.079)
**△CP**_**t**_	-0.006	-0.001	-0.00002	-0.006	-0.001	-0.00002
	(0.332)	(0.867)	(0.719)	(0.327)	(0.865)	(0.713)
**△IDR**_**t**_	0.007*	-0.046	-0.001	0.013**	-0.047	-0.001
	(0.067)	(0.384)	(0.167)	(0.015)	(0.382)	(0.168)
**△SVS**_**t**_	0.001***	-0.001	-0.00001	0.0001***	-0.001	-0.00001
	(0.000)	(0.735)	(0.838)	(0.000)	(0.732)	(0.843)
**△NCSKEW**_**t**_	53.590***	0.003	-0.00003			
	(0.000)	(0.197)	(0.269)			
**△DUVOL**_**t**_				53.683***	0.005	-0.00004
				(0.000)	(0.133)	(0.254)
**Constant**	53.590***	5.290***	-0.062***	53.683***	5.332***	-0.063***
	(0.00000)	(0.00000)	(0.00000)	(0.00000)	(0.00000)	(0.00000)
**Industry**	YES	YES	YES	YES	YES	YES
**Year**	YES	YES	YES	YES	YES	YES
**Firm**	YES	YES	YES	YES	YES	YES
**R** ^**2**^	0.163	0.178	0.042	0.163	0.178	0.042
**N**	21,472	21,472	21,472	21,472	21,472	21,472

Notes: For each variable, we report the regression coefficient and t-statistic (in parentheses) calculated

***,**, and * represent 1%, 5%, and 10% significant levels.

Changes in insurance participation and equity pledges are dependent variables, while changes in risk of collapse are explanatory variables. We seek to learn whether firms that have made equity pledges are less likely to collapse in the future, or whether firms with lower collapse risk are more likely to make equity pledges and engage in risky capital participation. We re-estimate the main model by considering the change in equity pledges as well as insurance participation from period t-1 to t (ΔIFPLEDGE_t_, ΔIFPLEDGE_t_*ΔIP_t_ and ΔIFPLEDGE_t_*ΔSP_t_) as dependent variables. The main explanatory variables stock price crash risk (ΔNSKEW_t+1_ and ΔDUVOL_t+1_) is the change from period t to t+1, and the control variables are the change from period t-1 to t. Panel B of Table 9 reports the results of the outcome tests for the reverse change. As predicted, we identified that the coefficients of ΔNSKEW_t+1_ and ΔDUVOL_t+1_ were not significant in all cases. Overall, the findings indicate that equity pledges and insurance participation influence the risk of future collapse, but that the risk of future collapse has no effect on equity pledges and insurance participation.

## 5. Conclusion and policy recommendations

This paper examines the effect of equity pledge on stock price crash risk from the perspective of insurance participation and reaches the following conclusions: first off, the risk of an enterprise’s stock price collapse under an equity pledge is low. This is primarily because the management of the company will improve internal corporate governance in order to qualify for an equity pledge and high leverage. It will also strengthen internal governance and raise the standard for earnings management, both of which will further encourage an improvement in the operating performance of the company. At the same time, from a market perspective, when a company pledges its equity capital, the market share will be reduced to a certain extent, causing the supply of shares to be less than the demand, thereby increasing the share price.

In addition, further research indicates that insurance participation can reduce the risk of collapse precipitated by an equity pledge through its regulatory effect. Following the intervention of insurance capital, the participating shareholders have a strong incentive to govern the company. They will subjectively strengthen internal governance management oversight in order to improve the company’s business performance and prevent a share price decline. Moreover, the intervention of insurance capital conveys good news to the market, resulting in a more positive market response, stronger bullish momentum, and boosted investor confidence. From the perspective of capital, it can increase market confidence. The company faces the risk of closing out and bursting when the pledge is inadequate, and the intervention of insurance capital can provide financial protection and support for the company when the liquidity of the company’s equity pledge is insufficient, and provide financial support when the risk of bursting is triggered when the closing line is reached. This has a regulatory effect and can effectively inhibit the share price’s downward trend; furthermore, the effect of reducing crash risk is more pronounced for private companies and those with a high shareholding ratio, and companies in manufacturing industry. This is because private companies have a higher demand for capital as their property rights are not state-owned, the degree of separation of powers and agency conflicts is greater in companies held by large shareholders, manufacturing companies usually have stable earnings and cash flow performance, and the financial support provided by insurers for equity pledges at risk can effectively reduce the risk of their collapse.

The conclusion of this paper demonstrates that the internal influencing mechanism of equity pledge to stock price crash risk from the perspective of insurance participation expands the current academic research on the economic effects of equity pledge, enriches the research perspectives, and offers a plausible explanation for resolving the risk of corporate capital liquidity. This paper puts forward the following suggestions: to reduce opportunistic speculation by company shareholders and enhance pledge efficiency; the market should first strengthen supervision and management of equity pledge, as well as establish different pledge management requirements and pledge rates for different types of companies. In addition, the closing line can be adjusted downward based on the company’s actual condition in order to reduce the risk of position bursting and the overall market risk. Secondly, during the process of market participation, insurance companies should effectively evaluate listed companies and purposely invest in high-quality listed companies, as opposed to investing for speculative purposes and conspiring with company managers to seize company profits. At the same time, market regulators should also effectively facilitate listed companies in enhancing the operational efficiency of the capital market and reducing market volatility. Last but not least, listed companies should improve information disclosure on equity pledge and internal management to reduce the likelihood of shareholders using this method to capture company profits, thereby mitigating the downside risk of share price due to illiquidity.

Due to the lack of data regarding insurance participation, the variables in this study are relatively limited. The depth and precision of studies on insurance firms’ investments are compromised by the limitations of insurer involvement in equity participation and the nature of equity participation included in the regression model as explanatory variables. In order to provide further policy suggestions to solve market problems and aid the smooth development of the capital market, future research can be closely related to the current development of the Chinese capital market by studying the impact of insurance company investment on market risk from various interest subjects according to market hotspots.
